# *In vitro* models of breast cancer bone metastasis: analyzing drug resistance through the lens of the microenvironment

**DOI:** 10.3389/fonc.2023.1135401

**Published:** 2023-04-11

**Authors:** Anaïs Lamouline, Simone Bersini, Matteo Moretti

**Affiliations:** ^1^ Regenerative Medicine Technologies Laboratory, Laboratories for Translational Research (LRT), Ente Ospedaliero Cantonale (EOC), Bellinzona, Switzerland; ^2^ Service of Orthopaedics and Traumatology, Department of Surgery, EOC, Lugano, Switzerland; ^3^ Department of Electronics, Information and Bioengineering, Politecnico di Milano, Milano, Italy; ^4^ Euler Institute, Faculty of Biomedical Sciences, Università della Svizzera italiana (USI), Lugano, Switzerland; ^5^ Cell and Tissue Engineering Laboratory, IRCCS Istituto Ortopedico Galeazzi, Milano, Italy

**Keywords:** drug resistance, breast cancer, bone metastasis, organ-on-a-chip, tumor microenvironment

## Abstract

Even though breast cancers usually have a good outcome compared to other tumors, the cancer can progress and create metastases in different parts of the organism, the bone being a predilection locus. These metastases are usually the cause of death, as they are mostly resistant to treatments. This resistance can be caused by intrinsic properties of the tumor, such as its heterogeneity, but it can also be due to the protective role of the microenvironment. By activating signaling pathways protecting cancer cells when exposed to chemotherapy, contributing to their ability to reach dormancy, or even reducing the amount of drug able to reach the metastases, among other mechanisms, the specificities of the bone tissue are being investigated as important players of drug resistance. To this date, most mechanisms of this resistance are yet to be discovered, and many researchers are implementing *in vitro* models to study the interaction between the tumor cells and their microenvironment. Here, we will review what is known about breast cancer drug resistance in bone metastasis due to the microenvironment and we will use those observations to highlight which features *in vitro* models should include to properly recapitulate these biological aspects *in vitro*. We will also detail which elements advanced *in vitro* models should implement in order to better recapitulate *in vivo* physiopathology and drug resistance.

## 1 Introduction

According to the World Health Organization, breast cancer is the most common cancer worldwide as of 2020, with the number of diagnoses having nearly doubled in the last two decades. Breast cancer is the second most deadly cancer amongst women, with mortality being especially high in countries with low income and in women of color (1–3). If the cancer is caught early enough, the treatments are usually very effective. However, breast cancer frequently metastasizes to bones, especially for the more advanced cases, rendering conventional treatments ineffective as metastases poorly respond to chemotherapy (4). Around 70% of the patients with advanced cancer will develop bone metastases (5).

It is now commonly accepted that breast cancer colonizes the bone according to the “seed and soil” theory proposed by Stephen Paget in 1889. According to this hypothesis, certain secondary organs such as the bone are better “soils” than others and are more at risk of developing metastases. Moreover, the primary tumor has the capacity to prepare the secondary loci for metastatic colonization, here the bone marrow, then cancer cells migrate towards this newly remodeled microenvironment as disseminated tumor cells (DTCs) through the vascular system. Once in the bone marrow, DTCs can stay quiescent for years before invading the surrounding bone (4). Awakened cancer cells interact with different bone cell populations (e.g. osteoblasts, osteoclasts, immune cells, mesenchymal stromal cells) to hijack and enhance the bone remodeling process in order to benefit their own growth. This process, called the vicious cycle, leads to what are known as osteolytic lesions, which are skeletal-related events linked to a loss of bone that causes debilitating pain for the patient. The bone is also considered a hub for further dissemination, as cancer cells in the bone get primed to further invade other organs (6).

To this date, no treatment to cure bone metastasis exists and only palliative care is available due to the inability to block the vicious cycle, as well as to drug resistance generated by the microenvironment (7). Indeed, it is now well known that the bone and the bone marrow exert a protective effect on cancer cells by shielding them from cytotoxic drugs or even increasing their aggressiveness (7–9). However, there is still a lack of knowledge regarding the specific role that the microenvironment plays in this process. In this context, the gold standard for pre-clinical drug testing (i.e. 2D assays in mono or coculture) cannot give enough information to accurately predict the effect of a drug. For example, 2D assays cannot recapitulate the bone remodeling process, even though the vicious cycle is one of the main reasons for the aggressiveness of bone metastasis. These models are also unable to properly recapitulate drug pharmacokinetics due to their lack of 3D perfusable blood vessels and extracellular matrix (ECM). The results obtained with traditional 2D assays are then generally validated in rodents, which have a different physiology compared to humans. Hence, the impact of the human tumor microenvironment is underestimated or even neglected during the first steps of the drug development process (10). For example, mouse/rat models have a vastly different immune system compared to humans, which affects the cancer progression as well as the metabolic reaction to anti-cancer drugs (11). Rodents also rarely form spontaneous metastases to bones (12). On top of that, animal models do not allow to easily perform parametric studies in high-throughput like removing or adding specific cell types to study a particular feature of the microenvironment. 3D human models hence represent a valuable tool to complement current pre-clinical drug testing approaches in order to more efficiently identify novel therapies. Indeed, these models can include various human cell types embedded in an ECM-like environment and allow for the high-throughput study of various biological mechanisms in a controlled and customizable setting, hence effectively predicting cancer cell behavior in response to drugs (13). The reader can refer to the following reviews for more details on the various existing 3D modeling techniques focused on oncology and drug screening (14–16). Even though it is currently not possible to completely bypass the use of animal models in drug testing, screening and analysis in every field, it is likely that the prevalence of complex *in vitro* models could reduce or even replace the requirement of systematic *in vivo* experiments in the next decades. For example, a recent study on liver toxicology showed that an organ-on-a-chip model outperformed conventional models, highlighting hepatotoxic reactions that were missed in animal models (17). Therefore, having a better comprehension of the interactions occurring in human tissues would most likely benefit the research community and potentially optimize the drug discovery pipeline.

In this review, we analyze the role of the bone microenvironment in the onset of drug resistance during the progression of breast cancer with the final goal to highlight which components and specifications 3D human bone models should include to properly recapitulate clinical observations.

## 2 Breast cancer bone metastasis and treatment

The development of bone metastases is associated with a poor prognosis: at this date, no cure exists and only palliative treatments are available. Moreover, the symptoms associated with bone metastases are very debilitating. They include severe pain, pathological fractures, nerve compression syndrome and hypercalcemia, all of them severely impairing the patient’s quality of life (18).

The process of bone metastasis formation is usually described with three steps, where the role of the microenvironment is tightly linked to the development of metastases. Firstly, the pre-metastatic niche is created. This process allows for the cancer cells to remotely influence the microenvironment of foreign tissues to facilitate future colonization by recruiting specific cancer-associated cells to remodel the ECM and create an immune-suppressive environment. Then, “educated” stromal cells facilitate the extravasation and attachment of DTCs (19). This process is driven by the interaction between cancer cell receptors with blood vessel surface ligands as well as with specific ECM proteins (e.g. laminins, fibronectin, vitronectin, osteopontin (OPN), bone sialoprotein). Indeed, these ECM proteins contain the arginylglycylaspartic acid (RGD) tripeptide that can be recognized by cancer cell surface proteins (e.g. integrins α_ν_β_3_ and α_4_β_1_, C-X-C chemokine receptor 4 (CXCR4), CD44) (20, 21). Cancer cells finally settle in the bone marrow niches (i.e. perivascular and endosteal), where they can remain dormant for years or even decades. During dormancy, these cells do not replicate and are able to evade the action of chemotherapy (22).

Once awakened, these metastases disrupt the bone remodeling process by enhancing bone resorption by osteoclasts and decreasing bone formation by osteoblasts. This process, called vicious cycle, is due to an increased bone resorption which releases growth factors that further stimulate metastasis growth (21, 23). Current therapies are generally initiated when overt metastases are already present and challenging to eradicate, mainly due to the onset of microenvironment-mediated drug resistance (9).

Breast cancer often becomes resistant to common therapies (24). For estrogen receptor positive (ER+) tumors, the first line treatment includes ER modulators or down-regulators. Currently, there are 6 drugs on the market targeting ER+ tumors. Most of these drugs are recommended to be used in combination with aromatase inhibitors, which are molecules that act directly on the estrogen production (24). Furthermore, 5 drugs are currently available for patients with human epidermal growth factor receptor 2 (HER2) positive tumors, all of them targeting either the extracellular or intracellular domain of this protein (24). Finally, 19 chemotherapy compounds have been approved for treatment, either as neoadjuvant or adjuvant drugs (i.e. before or after surgery resection, respectively), or for metastatic breast cancer. Though combinations of drugs are usually administered for an early-stage breast cancer, it appears that patients with metastatic cancer are usually administered the drugs one at a time, with 9 drugs most commonly prescribed (24, 25). All therapies and their effects are detailed in **Table 1**.

**Table 1:** Classification of currently available drugs targeting different breast cancer subtypes.

Type	Name of the drug	Name of molecule	Target	Effect	Side effects	Current use
**Endocrine therapy for HR+ tumors**	Novaldex (pill) Soltamox (liquid)	Tamoxifen	ER	Selective ER modulator	Blood clots, stroke, endometrial cancer	Most prescribed Early and advanced stage 5-10 year long treatment
	Faslodex	Fulverstrant	ER	Prevent oestrogen from biding to CCs -> ER down-regulators		ERBB2- No previous endocrine therapy Can be taken alone or with Ibrance
	Afinitor	Everolimus	FKBP12	mTOR inhibition -> restore sensitivity to ER treatments	Stomatitis, rash	Accepted by FDA with exemestane (aromatase inhibitor)
	Ibrance	Palbociclib	CDK4/6 inhibitors	Stop cell division	Lung, neutropenia (low count of white blood cells), blood clots	Used in combination with Faslodexinhibitor for advanced-stage or metastatic ERBB2-
	Kisqali	ribociclib			cardiotoxic, liver, lung issues, neutropenia	
	Verzenio	Abemaciclib			Blood clots & inflammation in the lungs	End of phase III for early high-risk to decrease the risk of relapse + combined with endocrine therapy

**Therapy for ERBB2+ (HER2+) tumors**	Herceptin	Trastuzumab	Extracellular domain of ERBB2	Slow/stop CC growth + alert immune system	cardiotoxic & lung issues	FDA approved for metastatic cancer
	Perjeta	Pertuzumab	Anti-ERBB2 Ab	Inhibits tumor growth	Cardiac dysfunction	Can be used in combination with trastuzumab
	Kadcyla	T-DM1 = herceptin + emtansine	chemo + ERBB2 target	action of emtansine without the extremely harmful side effects	cardiotoxic, lung and liver issues	FDA approved for advanced or metastatic
	Nerlynx	Neratinib	irreversible pan-HER chemical inhibitor	Inhibits tumor growth	renal impairement	FDA approved with capecitabine advanced or metastatic
	Tykerb	Lapatinib	small-molecule tyrosine kinase inhibitor	blocks ERBB2 inside the cell	small cardiotoxic issues	FDA approved with capecitabine or aromatase inhibitor Femara for advanced or metastatic

**Chemotherapy**	Adrucil	5-fluorouracil	DNA	antimetabolite - kills cancer cell during division	Neutropenia	Used with other chemotherapies Early or advanced
	Abraxane	Albumin-bound Taxane	Microtubules	Prevent cell division	Neutropenia, neuropathy	Used with other chemotherapies Advanced
	Adriamycin & Doxil	Doxorubicin Anthracycline	DNA	Block transcription + ROS production	cardiotoxic & leukemogenic	Early and advanced BC
	Cerubidine & DaunoXome	Daunorubicin Anthracycline	DNA	Block transcription + ROS production	cardiotoxic & leukemogenic	Early and advanced BC
	Cytoxan	Cyclophosphamide	DNA	Alkylating agent	Neutropenia	Advanced BC
	Ellence	epirubicin Anthracycline	DNA	Block transcription + ROS production	cardiotoxic & leukemogenic	Advanced or early BC
	Gemzar	Gemcitabine	DNA	antimetabolite	Neuropenia, anemia	Metastatic Used with other treatments
	Halaven	Eribulin	microtubules	Prevent cell division	Neutropenia, neuropathy, anemia	Metastatic BC that stopped responding to other treatments
	Ixempra	Ixabepilone	Tubulin	Prevent cell division		Metastatic Alone or with Xeloda
	Lynparza	Olaparib	PARP enzyme inhibitor	prevent PARP from reparing DNA damages	BM failure, leukemia, anemia, neutropenia	ERBB2-, BRCA1 or BRCA2+ Already treated with chemotherapy molecules
	Mexate, Folex, Rheumatrex	Methotrexate = amethopterin	DNA	antimetabolite		Advanced Used with other chemotherapy molecules
	Navelbine	Vinorelbine	microtubules	Vinca alkaloid	Neutropenia	Advanced
				blocks beta-tubulin		
	Novantrone	Mitoxantrone Anthracycline	DNA	Block transcription + ROS production	cardiotoxic & leukemogenic	Advanced BC
	Talzenna	Talazoparib	Inhibitor of PARP, PARP1 & PARP2 (that repair DNA)	prevent PARP from reparing DNA damages	BM failure, leukemia, anemia, netropenia, thrombocytopenia	ERBB2-, BRCA1 or BRCA2+ metastatic
	Taxol	Paclitaxel Taxane	antimitotic drug	Prevent cell division	Neutropenia	Most widely used anticancer drug
	Taxotere	Docetaxel Taxane	microtubules	Prevent cell division	Neutropenia, neuropathy	Advanced metastatic BC after other treatments
	Tecentriq	Atezolizumab	PD-L1 inhibitor	Allow T cells to kill the cancer cells by blockig the PD-1/PD-L1 interaction	Lung, liver, colon, hormone glands, cardiotoxic issues	FDA approved for metastatic TN , PD-L1+ with Abraxane
					Neutropenia, neuropathy	
	Thioplex	Thiotepa	DNA	Alkylating agent	neutropenia	Advanced BC with other chemo
	Xeloda	Capecitabine	DNA	antimetabolite Converted in cells in 5-FU		Metastatic BC that stopped responding to other treatments

**Other**	Avastin	Bevacizumab	VEGF	anti-angiogenic	cardiotoxic and kidney neutropenia	FDA approved with Taxol
	Paraplatin	Carboplatin	DNA	Platinum-based Damages the genetic material	kidney damage	advanced BC given in combination with chemotherapy

Bone metastasis can also be targeted using radiotherapy and immunotherapy. The latter treatment targets immune checkpoints such as programmed cell death protein-1 (PD-1), PD-ligand 1 (PD-L1) and cytotoxic T-lymphocyte–associated antigen 4 (CTLA-4). Immunotherapy has been approved in many cancers, sometimes at stage IV [such as metastatic bladder cancer (26)], and even as a pan-cancer treatment for the anti-PD1 antibody Pembrolizumab (as long as the cancer has microsatellite instability) (27). For more details, the reader can refer to these reviews (27–29). However, targeting bone metastases with immunotherapies has been challenging and to this date no systematic review has clearly highlighted the impact of immune checkpoint inhibitors. Indeed, despite the high number of immune cells in the bone marrow, most of them are still immature and cannot overcome cancer cell proliferation (30). Furthermore, the presence of inhibitory immune cells such as myeloid-derived suppressor cells severely impairs the eradication of metastases (30).

Multiple side effects can be observed upon treatment with both chemotherapy and immunotherapy. Regarding chemotherapy, most drugs focus on cells with a short replication time by targeting their DNA. Hence, a high number of normal cells with a high replication rate are also affected. Though the most common side effects are hair loss, soreness and digestive issues, chemotherapy can sometimes target very specific organs like the liver, kidneys, nerves and lungs, causing more debilitating symptoms such as urinary dysfunctions, pain, fatigue and dizziness, or even heart damage (31). Moreover, the higher the dose of chemotherapy, the higher the risk of side effects. For immunotherapy, the side effects commonly observed in chemotherapy (e.g. gastrointestinal diseases, mucositis or myelosuppression) are not or rarely observed. Generally, in around 10% of patients skin reactions and flu-like symptoms are noticed (32). The main side effects leading to casualties observed are colitis, pneumonitis, hepatitis and neurotoxic effects, however they tend to appear at a lower rate than with other treatments (33). Thus, having treatments that could target the microenvironment and increase the sensitivity of cancer cells to chemotherapy could not only impair cancer dissemination but also diminish the risk of side effects.

As of today, there are only two kinds of drugs specifically targeting bone metastases (i.e. Bisphosphates and Denosumab). These drugs are administered in combination with classic anti-cancer drugs. Bisphosphonates are molecules that inhibit the metabolic pathway of mevalonate, resulting in osteoclast apoptosis (34). The most effective and commonly used Bisphosphonate is Zoledronic Acid. This drug binds to hydroxyapatite and is released during bone digestion by osteoclasts. Osteoclasts then absorb it through endocytosis, where it inhibits the mevalonate pathway which is essential for post-translational prenylation of GTP-binding proteins. On the other hand, Denosumab is a monoclonal antibody that binds to RANK-L and inhibits the recruitment of osteoclasts (3). Both Bisphosphonates and Denosumab are palliative treatments that reduce pain and improve the quality of life of patients. However, their contribution to completely eradicate skeletal-related events (SREs) is limited. Moreover, these drugs are associated with side effects such as renal impairment and acute-phase reactions for Zoledronic Acid, and hypocalcaemia and osteonecrosis of the jaw for Denosumab (35).

Because anti-cancer treatments generally do not take into account the role of the bone microenvironment, with the exception of the partially effective Denosumab and Zoledronic Acid, it is emerging the concept that the next generation of therapies should combine traditional anti-cancer therapies with novel molecules targeting the other cell populations involved in the metastatic growth. In this scenario, the design of advanced *in vitro* 3D models recapitulating the metastatic bone microenvironment could significantly improve the identification of novel targets and the design of more effective combination therapies. The integration of these models with traditional animal studies could significantly increase the efficiency of the drug development pipeline and effectively identify mechanisms of drug resistance. For example, introducing 3D models as an effective high-throughput screening assay would help to determine with more efficacy which drugs or combinations have an impact on the disease progression. In addition, these models could be a useful tool to tailor the correct drug concentration, also considering potential side effects on the surrounding tissues (16). This approach would also allow to reduce the number of animals tested for each experiment and to limit the risk of testing molecules that were efficient in 2D but not in a 3D organ-specific context.

## 3 Role of the microenvironment in cancer cell proliferation and drug resistance

**Figure 1:**
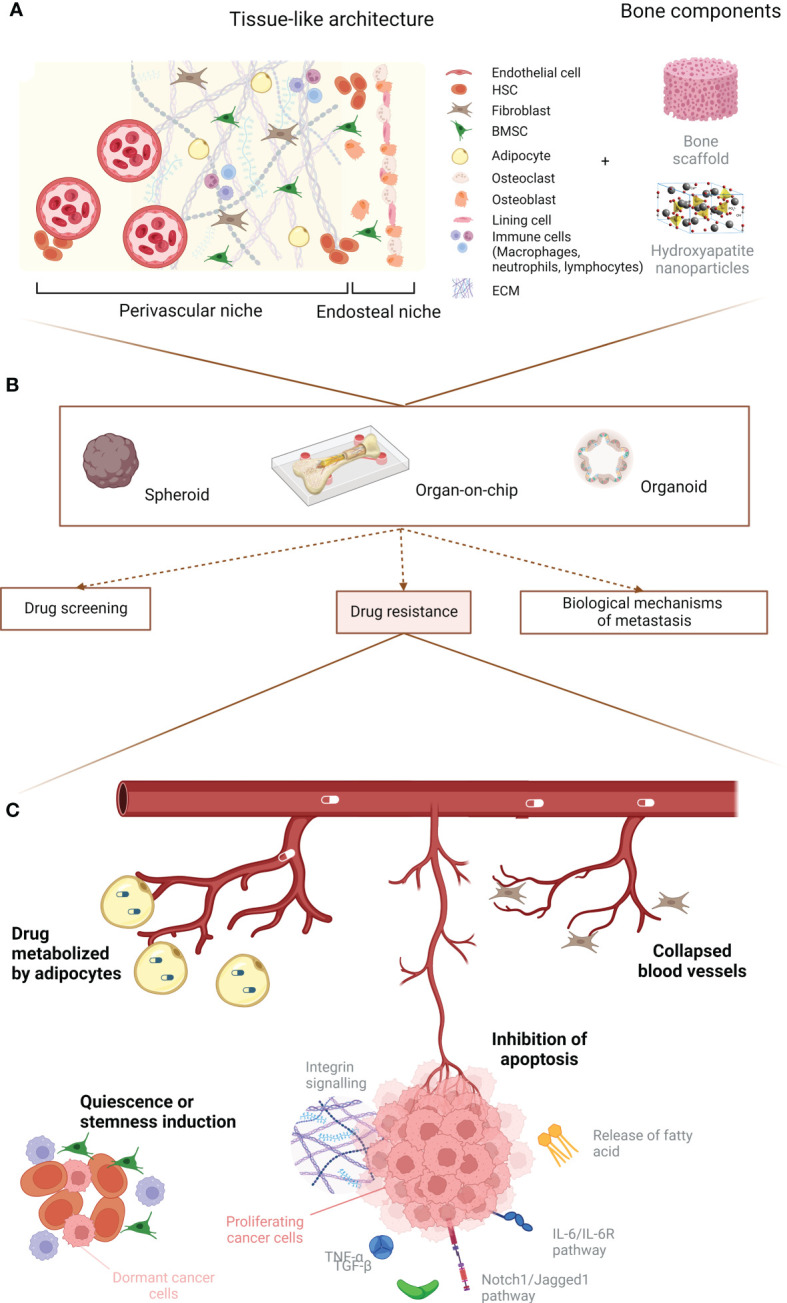
Graphical abstract. **(A)** Principal cellular players of the bone microenvironment. **(B)** Main uses for 3D *in vitro* models. **(C)** Summary of the predominant events leading to breast cancer drug resistance due to the microenvironment. Created with BioRender.com.

Multiple cell types are implicated in the bone metastasis process. Apart from osteoblasts and osteoclasts, additional stromal, vascular, immune and stem/progenitor cells appear to be essential for cancer cell homing, colonization, proliferation, quiescence and drug resistance. Here, we will describe how the bone microenvironment can influence cancer cell proliferation and drug resistance by detailing the role of cancer–associated fibroblasts (CAFs), macrophages, mesenchymal stromal cells, hematopoietic stem cells (HSCs) and adipocytes. Furthermore, we will analyze the contribution that the ECM and its remodeling have in boosting the growth of bone metastases. A graphical abstract of the role of the bone microenvironment in drug resistance is available in **Figure 1**. A summary of the main mechanisms, pathways and potential therapies in breast cancer bone metastases is compiled in **Table 2**.

**Table 2:** Biological mechanisms and pathways involved in metastatic breast cancer drug resistance.

Cell	General mechanism	Involved pathways (if known)	Potential therapies	Reference number
**Osteoblasts**	Decreased sensitivity to endocrine therapy due to loss of ER expression	IL-6/STAT3 direct cell-cell contact: gap junctions and Ca2+ signalling FGF2 & PDGF-DD induces EZH2-mediated reprogramming impacting Wnt and Notch TNFα/TNR1 MCP1/CCR2 (PI3K/Akt/mTor cascade)	Tocilizumab (anti-IL-R6 Ab) Sunitinib (PDGFR inhibitor) EPZ011989 (EZH2 inhibitor)	(36)
	Induction of stemness			(37)
	Inhibition of apoptosis	Jagged1/Notch1/p-53	15D11 (anti-Jagged1 Ab)	(38)
	Induction of dormancy	TNFα/TNR1 MCP1/CCR2 (PI3K/Akt/mTor cascade)	anti-TNR1 or anti-CCR2 antibody	(39)
**MAFs**	Supression of T cell function	Prostaglandin E2, TGF-β, VEGF		(22, 40)
	Collapse of blood vessels -> decreased drug delivery	Increased collagen and hyaluronan production	Losartan (TSP-1 inhibitor) Angiostin signalling blockade	(41)
	Induction of stemness	IL-6	See above	(42, 43)
**TAMs**	Decrease endocrine therapy sensitivity	secretion of CCL2 activating the PI3K/Akt/mTor signalling pathway		(44)
	Induction dormancy	GJIC		(45)
**MDSCs**	Supression of T cell function	Arginase expression	See paper from Chesney et al	(46)
**Neutrophils**	Inhibition of apoptosis	TNF-α/CXCR2 S100A8/9	TNF-α antibody CXCR2 blockers	(47)
**MSCs**	Supression of T cell proliferation	Secretion of TGF-β, hepatocyte growth factors, IDO, PGE2, nitric acid suppression of Stat5 phosphorylation	antibodies against TGF-β or hepatocyte growth factor inhibitors of IDO, prostaglandin or NOS	(48)
	Induction of dormancy	GJIC and transfer of miNRA targeting CXCL12	target of miRNA target of GJIC (ex: H89)	(49, 50)
	Inhibition of apoptosis	polyunsaturated fatty acid (PIFA) released upon platinium-based treatment	blockade of PIFA-producing enzymes	(51)
		TGF-β		(52)
**Adipocytes**	metabolizes and deactivates chemotherapy molecules			(53, 54)
**Microvascular endothelium**	Inhibition of cytostatic effect	Secretion of integrins (β1 and αvβ3) Von Willebrand Factor VCAM1	Antibodies against integrins β1 and αvβ3	(55)
**ECM**	Inhibition of apoptosis	β1 and αvβ3 integrins PI3K/Akt pathway	Antibodies against integrins β1 and αvβ3	(56, 57)

Mechanisms and pathways are divided based on specific cell populations of the bone microenvironment.

### 3.1 Osteoblasts

Together with osteoclasts, osteoblasts are known to play a role in the proliferation of bone metastasis since they are involved in the vicious cycle. Osteoblasts tend to show decreased activity and send signals for the recruitment of osteoclasts, leading to enhanced bone loss (58). Osteoblasts can also contribute to the survival of cancer cells in the bone, as regulation of calcium intake by cancer cells cannot be done without the presence of osteogenic cells. Indeed, cancer cells cannot efficiently absorb calcium from the microenvironment and a direct cell-cell connection using Connexin 43 is used to transfer calcium ions from osteoblasts to cancer cells (59). In this context, gap junction inhibitors like MEFL or CBX do present a positive outcome in inhibiting this interaction, although as a side effect they are known to increase the vertebral curvature. On the other hand, two already approved drugs (i.e. Danusertib and Arsenic Trioxide) were shown to effectively block this survival mechanism.

Osteoblasts also have a role in breast cancer drug resistance. For example, osteoblasts are known to produce high levels of interleukin (IL)-6 (60), which correlates to Tamoxifen resistance. Inhibition of IL-6Rα with the FDA-approved antibody Tocilizumab was able to sensitize resistant ER+ breast cancer cells to Tamoxifen both *in vitro* and *in vivo* (36). Moreover, direct (through gap junction and calcium signaling) and indirect (through fibroblast growth factor 2 (FGF2) and platelet-derived growth factor-DD (PDGF-DD) secretion) interactions between cancer cells and osteogenic cells leads to a reduction in estrogen receptors. This process translates into a decreased sensitivity of ER+ breast cancer cells to endocrine therapy, which involves an enhancer of zeste homolog 2 (EZH2)-mediated reprogramming that also seems to induce stemness in breast cancer bone metastasis (37). Osteoblast lineage cells also tend to protect cancer cells against chemotherapy in the bone through the overexpression of Jagged1, which interacts with the Notch1 receptor on cancer cells and circumvents apoptosis by affecting the p53-regulated apoptotic pathway. Injection of a Jagged1 inhibitor (such as an anti-Jagged1 monoclonal antibody, here 15D11) in mice improved the chemosensitivity of cancer cells, making it a promising candidate for targeting the bone microenvironment (38). Osteoblasts can also protect breast cancer cells from chemotherapy by inducing a dormancy state, notably due to the secretion of cytokines such as tumor necrosis factor α (TNFα) and monocyte chemoattractant protein-1 (MCP1). The first one modulates the Fas-associated death domain protein (FADD)/tumor necrosis factor receptor type 1-associated death domain protein (TRADD) by biding to the TNF receptor 1 (TNFR1), while MCP1 binds to C-C chemokine receptor type 2 (CCR2) and induces the phosphatidylinositol 3-kinases/protein kinase B/mammalian target of rapamycin (PI3K/Akt/mTOR) cascade (39).

Osteoclasts represent the counterpart of osteoblasts in the bone remodeling process, since they play a very important role in the vicious cycle and thereby in the aggressiveness of the tumor (58). However, their role in drug resistance has not yet been studied.

### 3.2 Metastasis-associated fibroblasts

Metastasis-associated fibroblasts (MAFs) (also called CAFs for cancer-associated fibroblasts when talking about cancer in general) also seem to have an important role on the metastatic proliferation of breast cancer cells into bone. MAFs can derive from multiple cell lines including mesenchymal stromal cells (MSCs) in the bone marrow (61), adipocytes, pericytes and even endothelial cells (62). Furthermore, inflammatory modulators such as interleukins, direct contact with cancer cells and physical changes in the ECM architecture contribute to the differentiation of MAFs (62). These activated cells produce growth factors (e.g. hepatocyte growth factor (HGF), transforming growth factor β (TGF-β), stromal derived factor 1/C-X-C motif chemokine ligand 12 (SDF-1/CXCL12), vascular endothelial growth factor (VEGF), insulin-like growth factor 1 (IGF-1)), interleukins, matrix metalloproteinases (MMPs) and exosomes which promote primary tumor and metastatic growth (63).

The presence of MAFs in the tumor microenvironment has been shown to cause chemoresistance through mechanisms that are so far poorly understood. A study showed that a few key players (e.g. retinoic acid receptor β (RARβ), peroxisome proliferator-activated receptor β and δ (PPARβ/δ), vitamin D receptor (VDR), glucocorticoid receptor (GR) and androgen receptor (AR) in the context of skin cancer) seem to be involved in both cancer aggressiveness and drug resistance. Moreover, targeting nuclear receptors that modulate the expression of those proteins led to a decreased drug resistance to cisplatin (64).

MAFs also tend to suppress the normal function of immune T cells in the microenvironment through immunosuppressive factors (e.g. prostaglandin E2, TGF-β, VEGF). Moreover, MAFs secrete pro-inflammatory cytokines including IL-6 that help to recruit tumor-associated macrophages (TAMs) and promote their transition from M0 to immune-suppressive M2 (22, 40).

MAFs are also known for stimulating angiogenesis, mostly through secretion of VEGF, platelet-derived growth factor C (PDGF-C) and IL-6 (22, 65). At the same time, MAFs can induce the collapse of existing blood vessels due to increased matrix stiffness, leading to hypoxia and to the proliferation of more aggressive metastatic clones. This effect can be reduced by a blood-pressure medication, Losartan, that decreases both the amount of collagen and hyaluronan causing vessel compression, and MAF activation (41). Importantly, this lack of blood vessels also leads to a decrease in drug delivery, enhancing the appearance of drug resistance (41, 66). Importantly, multiple studies also place MAFs as important protagonists in drug resistance, although not focused specifically on breast cancer. These studies highlight the role of MAFs in the secretion of IL-6 which enhances drug resistance by increasing the endothelial to mesenchymal transition (Endo-MT) and the generation of cancer stem cells (42, 43). Finally, upon chemotherapy administration it was shown that normal stromal fibroblasts tend to switch from an aerobic to glycolytic metabolism, correlating with a transformation of stromal fibroblasts into MAFs (67). Although not directly related to drug resistance, this study highlights how conventional therapies can alter the microenvironment and paradoxically enhance the metastatic dissemination.

Even though no treatment options have been tested in the specific context of breast cancer, a few therapeutic strategies have been evaluated to specifically target these aberrant fibroblasts. For example, in pancreatic adenocarcinoma, administering both Nab-paclitaxel and Gemcitabine helped in reducing the amount of CAFs in patients. In non-small-cell lung cancer, combining Paclitaxel with a tyrosine kinase inhibitor (i.e. Nindetanib, targeting VEGF, FGF and PDGF) impaired the interactions between cancer cells and CAFs. Finally, targeting TGF-β with monoclonal antibodies like Fresolimumab or Galunisertib seems to be a promising treatment strategy in various cancers (68).

### 3.3 Immune cells

#### 3.3.1 Macrophages

Macrophages can be polarized by cancer cells towards an M2, anti-inflammatory phenotype. These M2 macrophages make up the majority of the so-called tumor associated macrophages (TAMs). These cells can constitute up to 30-50% of the tumor mass in skeletal metastases and are associated with a poor prognosis as they promote an immunosuppressive environment (69). This polarization towards an M2 phenotype tends to be enhanced by a positive feedback loop where TAMs secrete C-C motif chemokine ligand 2 (CCL2) that activates the PI3K/Akt/mTOR signaling pathway in cancer cell. This pathway tends to increase the resistance to ER modulators. Resistant cells then secrete TNF-α that activates TAMs and induces their M2 polarization (44).

TAMs seems to have a role in treatment resistance, as macrophage inhibitors (e.g. colony stimulating factor 1 (CSF-1) inhibitor) prior to chemotherapy have been shown to enhance the response to treatment in mammary and cervical cancer (70, 71). Similarly, treatment of mice with Cyclophosphamide (i.e. chemotherapy with immune-suppressive properties) combined with CSF-1 inhibitor reduced macrophage recruitment to the tumor and reversed chemotherapy resistance (44).

Finally, although not many studies consider the specific role of TAMs in the bone or bone marrow microenvironment, it seems that TAMs in the bone marrow are able to induce breast cancer cell dormancy by using GJIC, a mechanism used to evade chemotherapy which only targets rapidly-proliferating cells (45).

#### 3.3.2 Myeloid-derived suppressor cells

Myeloid-derived suppressor cells (MDSCs) are immature myeloid cells that later differentiate into macrophages, dendritic cells or granulocytes. However, in presence of cancer cells there can be an accumulation of immunosuppressive MDSCs, leading to a suppression of both innate and adaptive immune response (46). In bone metastasis, MDSCs are shown to contribute to immunotherapeutic resistance by inhibiting antitumor T cell proliferation and cytotoxic activity. They also promote the expansion of protumorigenic T regulatory cells, thus dampening the host immune response against the tumor, which in turns promotes angiogenesis, tumor invasion and metastasis (46). The many preclinical and clinical MDSC inhibitors are detailed elsewhere (46).

One study highlighted the role of MDSCs in drug (i.e. 5-FU) resistance in the case of hepatocellular cancer. This effect was mediated by the secretion of IL-6 (72), which is also known to increase drug resistance in metastatic breast cancer (36, 42, 43, 73). Moreover, in multiple myeloma (i.e. a bone marrow cancer) it has been shown that MDSCs had a direct influence on resistance to Doxorubicin and Melphalan due to the release of soluble factors (74). However, as these soluble factors have not been identified, it would be interesting to determine if they would induce the same effect on other cancer cell types.

#### 3.3.3 Neutrophils

Neutrophils are cells that tend to be cytotoxic towards cancer cells during the first stages of the metastatic process, but that effect appears to be lost as tumor progresses. Indeed, neutrophils create an immunosuppressive action at the late stages of the cancer (75). Neutrophils are also known to help with the generation of the pre-metastatic niche by enabling circulating tumor cell (CTC) lodging at the metastatic sites. Mature neutrophils are shown to accumulate in the metastatic site even before the arrival of cancer cells, thereby helping to create the pre-metastatic niche by inducing an *in-situ* vascular remodeling and stimulating metastasis initiation (76).

As described with MAFs, neutrophils can also have a pro-tumorigenic effect post-chemotherapy treatment. Indeed, release of TNF-α by bone marrow derived cells in response to chemotherapy treatment has been observed, which leads to the activation of Nuclear factor kappa B (NFϰB) and secretion of CXCL1/2 by cancer cells. These signals attract neutrophils to the tumor site where they produce S100A8/9 enhancing cancer cell survival. The hypothesis of anti TNF-α antibodies or CXCR2 blockers as a treatment option is currently being investigated (47).

#### 3.3.4 T-cells

T cells are a group of immune cells critical to the adaptive response to pathogens and aberrant cell proliferation. They are divided in two main categories, CD4+ (or Th, for T helper cells) which modulate the activity of other immune cells, and CD8+ (or CTLs, for cytotoxic T lymphocytes) which can induce cell death (77). In cancer, immunosuppressive mechanisms are put in place, both by cancer cells and the tumor microenvironment to hinder the effectiveness of T cells (78). It has been shown that the presence of T cells in the tumor site increases the chance of immunotherapy response (79). However, a subcategory of T cells called regulatory T cells (T_regs_), known to suppress the immune response (80), seems to have an impact on cancer cell proliferation. Indeed, tumor growth is promoted by the infiltration of leukocytes towards the stromal compartment of the bone marrow. This process seems to be partially mediated by the recruitment of T_regs_ (81). One of the treatments currently in place to reduce T cell dysfunction and exhaustion, two mechanisms that contribute to the immunosuppressive environment of the tumor, is PD-1/PD-L1 blockade. Indeed, cancer cells overexpress PD-L1, leading to an imbalance of the ratio of PD-1high CTLs and PD-1low CTLs, with a higher number of PD-1high CTLs in cancer patients compared to healthy ones that causes dysregulation and exhaustion of CTLs. However, this blockade might stimulate the expression of PD-1 in T_regs_, which causes further immune resistance (79).

In prostate cancer, a study showed that the presence of CD4+ T cells in the tumor site promotes chemotherapy resistance (specifically to Enzalutamide and Doxorubicin) through the C-C motif chemokine ligand 5 (CCL5) signaling pathway (82), known to activate the Signal transducer and activator of transcription 3 (STAT3). This pathway could also be involved in breast cancer chemoresistance (83).

### 3.4 Mesenchymal stomal cells

Mesenchymal stromal cells (MSCs) are known to have an important role in bone metastasis development. By being the progenitors for osteoblasts, MSCs play a critical role in osteoblast proliferation, bus also in osteoclastogenesis, angiogenesis and immunosuppression (84–86). MSCs are also essential for HSC maintenance and thus contribute to the survival of the bone marrow niche. When it comes to bone metastases, multiple studies point towards a pro-tumorigenic role of MSCs, mainly *via* activation of MMPs which promote angiogenesis, stimulate epithelial-mesenchymal transition (EMT) and suppress the immune response, notably T-cell proliferation through secreted molecules or free radicals (48, 87). Moreover, MSCs may play a role in preserving the self-renewal ability of cancer cells as they do with HSCs, thus favoring the establishment of a tumor niche with long-term proliferative potential (20). This ability is believed to be effective on a small subset of breast cancer cells that show high expression of the pluripotency marker *OCT4*. This subset has been shown to create gap junctional intercellular communications (GJICs) with bone marrow stromal cells, a process that allows the transfer of quiescence-promoting miRNA, thus inducing dormancy and subsequently chemotherapy evasion and cancer relapse even after high doses of chemotherapy (88). This effect seems to be due to miRNA targeting CXCL12 in cancer cells, leading to decreased levels of CXCL12 and lower proliferation. These miRNAs could be a therapeutic target (49). In order to more accurately define and target these GJICs, a study showed that these connections were mediated by the protein kinase A (PKA) enzyme, whose activation could be induced or inhibited (i.e. using LY294002/LY303511 and H89, respectively) (50).

DTCs also use chemokine gradients in the bone marrow and adhesion molecules expressed by the HSC niche (e.g. CXCL12) that are believed to be crucial for HSC homing and survival (89). It is believed that cancer cells can acquire chemotherapy resistance by using an HSC-like state of dormancy to avoid drugs targeting rapidly-proliferating cells. Both cancer cells and HSCs use similar pathways to induce dormancy, such as the CXCL12/CXCR4 axis. The implication of CXCL12 in dormancy is controversial: on one hand, it is known to promote HSC self-renewal and pool maintenance, either by supporting their self-division or potentially by inhibiting their cycling status (90). On the other hand, CXCL12 is mostly known to activate key survival signaling pathways upon biding to CXCR4, such as the PI3K/Akt, the mitogen-activated protein/extracellular-signal-regulated kinases (MAPK/ERK) and the Janus kinase/signal transducer and activator of transcription (JAK/STAT). Contradicting studies have shown either an overexpression and a dowregulation or CXCR4 in breast cancer bone metastasis, both pointing to a proliferative role of the CXCL12/CXCR4 axis (91–93). The mechanism leading to an avoidance of cancer cells rapid proliferation by CXCL12 and, thus chemotherapy treatment (94) is, so far, poorly understood. It has also been found that this dormancy is related to the Notch2 pathway, as blocking this signal resulted in a mobilization of previously dormant breast cancer cells (95).

The role of MSCs in breast cancer drug resistance can be induced by chemotherapy itself. For example, platinum-based chemotherapy, commonly used in triple negative breast cancer (TNBC) (96), can induce resistance mechanisms due to the reaction of MSCs to platinum. Indeed, upon platinum stimulation MSCs can release poly-unsaturated fatty acids, which induce broad-spectrum resistance to chemotherapeutic agents (51). In other cancers, such as leukemia, MSCs can protect cancer cells from further chemotherapy-induced apoptosis through the activation of the TGF-β pathway (52).

### 3.5 Adipocytes

Although previously underestimated, increasing interest is growing around the contribution of the adipogenic niche in bone metastasis. Indeed, elderly patients are characterized by an increase of bone marrow adipogenic niche when aging (97). Furthermore, direct cellular interactions occur between cancer cells and adipocytes, for instance through adipose-derived leptin and IL-1β (65). More importantly, bone marrow adipocytes alter the metabolism of cancer cells, stimulate cell adhesion, colonization and proliferation, and promote resistance to chemotherapy through various adipokines (98). For example, it has been shown that metastatic breast cancer cells tend to migrate more towards a medium enriched with leptin, a protein released by adipocytes (99). Moreover, lipids arising from adipocytes have been demonstrated to increase tumor growth and invasiveness by increasing the expression of fatty acid binding protein 4 (FABP4, i.e. fatty acid chaperone that is involved in glucose and lipid metabolism), heme oxygenase 1 (HMOX) and IL-1β (100). It has also been shown that cancer cells can hijack adipocytes and transform them into cancer-associated adipocytes (CAAs), that overexpress IL-6 and other pro-tumor cytokines (101). Regarding anti-cancer treatments, a major concern around adipocytes is that the adipose tissue is known for metabolizing and thus deactivating chemotherapy drugs (53). Co-culture of adipocytes and cancer cells in presence of Doxorubicin enhances the capacity of cancer cells to store the drug in vesicles instead of in the nucleus, which further increased the resistance of cancer cells (54). Moreover, in specific cases of ovarian cancer metastases, adipocytes were reprogrammed towards a more catabolic state and secreted free fatty acids that were used by cancer cell to generate ATP, hence conferring chemoresistance (102). Currently, it is still unknown if these mechanisms also happen in bone metastases from breast cancer.

### 3.6 Microvascular endothelium

DTCs mostly reside in the perivascular niche, which is a region in close proximity to blood vessels. A specificity of the bone marrow is indeed the capacity of its blood vessels to express adhesive molecules (e.g. P-selectin, E-selectin, intracellular adhesion molecule 1 and vascular cell adhesion molecule 1) without the requirement of stimulation by inflammatory cytokines, contrary to other tissues. These molecules interact with cancer cells and facilitate their adhesion (103). Within this niche, the distance between a cancer cell and a blood vessel is almost 30 times smaller than the average distance between an osteoblast and a blood vessel. Following chemotherapy treatment, the distance between cancer cells and blood vessels is even reduced suggesting that cells located closer to blood vessels are resistant to the treatment. This resistance seems to be directly linked with the expression of integrin β_1_ and integrin α_v_β_3_ on cancer cells, since inhibiting these integrins with specific antibodies induced an increased sensitivity to Doxorubicin (55). In this scenario, it seems that integrin α_v_β_3_ protects DTCs from chemotherapy through signaling triggered by endothelial Von Willebrand Factor (VWF). In addition, DTC chemoresistance is driven by vascular cell adhesion molecule 1 (VCAM1). This endothelial surface molecule is an integrin α_4_β_1_ ligand along with other endothelial-derived integrin α_4_β_1_ ligands. Combined treatment with antibodies targeting both integrins β_1_ and α_v_β_3_ leads to a higher percentage of DTC cells sensitized to Doxorubicin both *in vitro* and in mice (55).

In lymphomas, which frequently metastasize to the bone marrow, a vicious cycle between B cells and endothelial cells occurs. FGF-4 is secreted by lymphoma cells, leading to an activation of fibroblast growth factor receptor FGFR-1 on endothelial cells and to an upregulation of Notch ligand Jagged1. As a consequence, lymphoma cells increase their aggressiveness, invasiveness and chemoresistance (104). It is important to note that breast cancer cells also use the Jagged1/Notch pathway to promote bone metastases (105), thus a focus on FGFR-1 expression in breast cancer bone metastases could unveil new answers in drug resistance mechanisms.

### 3.7 Tissue non-specific chemotherapy-induced resistance

Chemotherapy itself can act as a metastasis-inducer. For example, a chemotherapy treatment on the initial primary breast tumor can select chemoresistant clones that could form metastases, leading to secondary tumors already resistant to the first line of chemotherapy (106). Moreover, a few *in vitro* studies demonstrated that preoperative/neoadjuvant as well as post-operative/adjuvant chemotherapy can induce metastases. This phenomenon could be due to the induction of tissue damage that the body repairs through the secretion of cytokines that also promote the generation of resistant clones (107). For example, Paclitaxel activates the toll-like receptor 4 (TLR4), which is present on macrophages to recognize polysaccharides and it is also expressed by breast cancer cells. This activation tends to exacerbate a pro-inflammatory microenvironment, leading to angiogenesis and cancer cell invasion. Paclitaxel can also promote an EMT-like phenotype in cancer cells (i.e. decreased E-cadherin expression and increased formation of invadopodia), thus enhancing the likelihood of metastases. Chemotherapy can also increase the risk of CTCs invading secondary tissues by inducing the release of platelet-derived microvesicles that bind to the CTC surface and facilitate their attachment to the endothelium. These “coated” CTCs are also more protected against immunological destruction by natural killer (NK) cells (107).

### 3.8 ECM

Cancer cells expressing α_v_β_3_ integrin, which is often upregulated in breast cancer, bind to the ECM components fibronectin and osteopontin, which along with vitronectin are highly expressed in bone marrow. In epithelial cancers it has been shown that adhesion through α_v_β_3_ integrins leads to chemotherapy resistance. A similar effect has been reported with β_1_ integrins, most likely due to a protective effect of the nuclear response to DNA-targeting agents (56). Moreover, expression of β_1_ integrins also showed a drug resistance effect on Paclitaxel and Vincristine, two molecules targeting the microtubules. The signaling pathways activated through β_1_ integrin ligation induce an inhibition of cytochrome c release and activation of the PI3K/Akt pathway, reducing the expected apoptosis effect of the chemotherapeutic agents (57). Even though the specific role of the ECM in resistance is still yet to be fully understood, it seems that targeting elements of the cell-matrix interaction (e.g. integrins) through novel mechanobiological therapies could help to increase the sensitivity of cancer cells to chemotherapy treatment.

## 4 *In vitro* models of bone metastasis to analyze drug resistance

It is now becoming evident that the microenvironment plays a non-negligible role in the homing, survival, proliferation and drug resistance of cancer cells. In the bone, multiple cell types induce various pathways that help the cells to avoid or resist chemotherapy treatment. For instance, osteoblasts, MSCs or even ECM proteins can directly influence the apoptosis pathway in cancer cells, while TAMs, MSCs or HSCs allow cancer cells to adopt a dormancy behavior. Furthermore, MAFs, MDSCs or MSCs can decrease the efficacy of immunotherapy by suppressing T cell function. Other cell populations can have a more direct impact on drug distribution: adipocytes metabolize and eliminate chemotherapy molecules from the tissue, while MAFs induce the collapse of blood vessels, hence decreasing the availability of the drug. Overall, it is clear that drug screening without the presence of a microenvironment significantly decreases the likelihood of recapitulating the actual action of the drug in humans.

However, so far drug screening assays have traditionally been performed on simplified 2D cultures of cancer cell lines. Animal and clinical studies have highlighted that the local microenvironment is a key mediator of the drug resistance observed in patients. Hence, the next generation of pre-clinical models should focus on the successful integration of organ-specific, physiological-like microenvironments to overcome limitations of conventional systems (108–111). Indeed, *in vitro* models represent a useful tool to identify earlier potential drug resistance mechanisms during the drug development pipeline or clarify basic mechanisms that cannot be quickly identified with animal studies.

In this scenario, introducing a 3D microenvironment without a complex cellular composition has already proved to be effective in modeling biological processes or drug response. Kim et al. proposed a microfluidic device where MCF-7 breast cancer cells were able to aggregate into spheroids and showed that the drug sensitivity of the cancer cells in the spheroids was decreased compared to 2D monolayers (112). Increasing the level of complexity, some models included a very basic 3D microenvironment. For instance, co-cultures of cancer-associated fibroblasts with breast cancer cells showed higher drug resistance compared to a 3D monoculture of cancer cells (113). The authors hypothesize that this drug resistance might be due to both reduced drug penetration in spheroids (compared to a monolayer) and to intercellular contacts activating cell survival pathways such as PI3K/Akt, NF-κB and STAT3. However, the specific pathways involved were not investigated. These results confirm that 3D models populated with a simplified microenvironment can help to decipher differences in drug sensitivities due the presence of both the 3D matrix (i.e. the interactions with the ECM and the decreased drug diffusion compared to 2D), as well as the surrounding cells.

**Figure 2:**
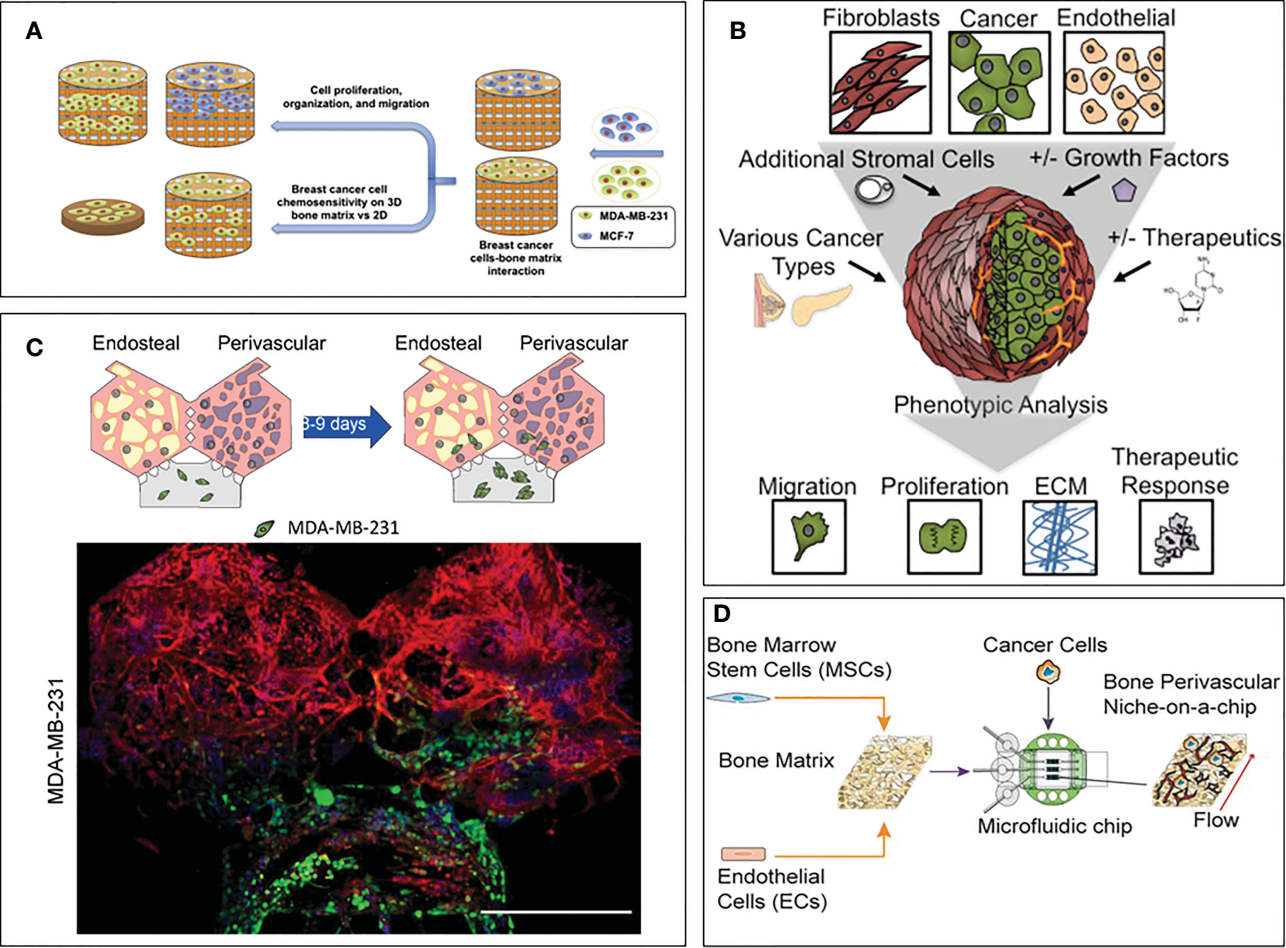
3D models focusing on drug sensitivity in breast cancer bone metastasis. **(A)** Model by Zhu et al. (114). Breast cancer cells and bone marrow MSCs were seeded on a bone matrix and showed drug resistance in presence of the microenvironment compared to the 2D culture. **(B)** Model by Langer et al. (119). Breast cancer cells were seeded with fibroblasts, endothelial cells, adipocytes and bone marrow MSCs. Increased cancer cell aggressiveness and drug resistance were observed compared to the 2D control. **(C)** Model by Glaser et al. (121). Breast cancer cells were seeded with bone marrow MSCs, osteoblasts, HSCs and endothelial cells. Differences in cell behavior were observed when breast cancer cells were co-cultured with bone and stromal cells compared to fibrin only hydrogels. **(D)** Model by Marturano-Kruik et al., (120). Cancer cells showed drug resistance when exposed to interstitial flow.

A few biofabricated 3D models specifically focused on studying the drug resistance of breast cancer due to the bone microenvironment compared to a simplified control (often 2D). Zhu et al. created a bone model using a biomimetic bone matrix seeded with human bone marrow MSCs and MDA-MB-231 or MCF-7 breast cancer cell lines (114). The study highlighted a higher drug resistance when the 3D microenvironment was subject to an anti-cancer treatment with 5-FU compared to 2D culture. However, the authors did not propose an analysis of the pathways involved in the observed resistance, even though they hypothesize that reduced drug diffusion and altered transporter expression due to cell-matrix interactions could play a role. Similarly, Kar and co-authors created a co-culture model on a 3D polycaprolactone/hydroxyapatite PCL/HAP clay scaffold with MSCs and either MDA-MB-231 or MCF7 breast cancer cell lines (115). The authors compared the sensitivity to Paclitaxel of these cell lines in their 3D co-culture and 2D monoculture showing a higher resistance in 3D. This resistance was correlated with an up-regulation of STAT3, leading to the overexpression of B-cell lymphoma 2 [Bcl-2, known to have an increased expression in chemoresistant cells (116)] or multidrug resistant protein 1 (MRP1) and ATP Binding Cassette Subfamily G Member 2 (ABCG2) [known to be upregulated in multiresistant cells, meaning cancer cells resistant to multiple chemotherapeutic drugs with different structures and mechanisms of action (117, 118)]. Likewise, Langer and co-authors assessed the effect of the microenvironment on drug sensitivity (119). The proposed model included fibroblasts, endothelial cells, adipocytes and bone marrow MSCs, which have been shown to have a detrimental effect on drug treatment. Using MCF-7 breast cancer cell line, the authors showed that the microenvironment not only contributed to the aggressiveness of the metastatic process, but also to drug resistance. Indeed, the concentrations of Doxorubicin and Paclitaxel were respectively 20 times and 5,000 times higher in the 3D model than in the 2D control to achieve the same level of cancer cell mortality. The study also showed that part of this effect was due to a paracrine influence of fibroblasts by using a fibroblast-conditioned medium in a 3D cancer cell monoculture, where the authors still observed a lack of therapeutic response. The authors concluded that secreted factors from fibroblasts were able to induce resistance to mTOR inhibition. The device proposed in this study is a great example of the usefulness of 3D models, both for the field of drug screening but also for deciphering the molecular pathways involved in drug resistance. Finally, another bone model composed by bone marrow MSCs and endothelial cells on a decellularized bone matrix (120) showed a higher drug resistance of breast cancer cells when the whole system was subject to an interstitial flow compared to static conditions. This phenomenon is explained by the better maturation of the vasculature (compared to the static condition), leading to less proliferative cancer cells. This particular feature makes this model a great tool to study dormancy in the bone niche. This finding highlights the importance of recapitulating both the cellular complexity and the biophysical stimuli characterizing the bone microenvironment in order to fully reproduce *in vivo* mechanisms. These models are summarized in **Figure 2**.

To date, very few models are focusing on the effect of the bone microenvironment on drug resistance, especially considering breast cancer. Even though the field of drug testing in 3D models is still at its infancy, many researchers are starting to highlight the benefits of including complexity in the drug screening process. Since the immune system plays a significant role in terms of metastatic proliferation and drug resistance, a few models included immune cells when studying breast cancer bone metastases. However, these models are not yet considering drug resistance mechanisms. For example, Crippa et al. designed a breast cancer bone metastasis model embedding endothelial cells, bone marrow MSCs, osteoblasts, fibroblasts and neutrophils in two separate chambers connected by perfusable vessels (122). This study showed a higher recruitment of neutrophils when breast cancer cells were present in the device, as well as a higher cancer cell mortality due to neutrophil attack. However, the authors did not perform any drug treatment through the model. It would have been interesting to use that model to test the influence of the immune system on drug sensitivity, especially with neutrophils which are known to have both an anti- and pro-tumorigenic effect. In another study, the immune system was introduced as bone-resident macrophages (123), which were co-cultured with breast cancer cells, endothelial cells, osteoblasts and osteoclasts. The model was then challenged with Doxorubicin or Rapamycin. The authors showed that the bone microenvironment protected cancer cells from both treatments, hence more closely mimicking what is observed *in vivo* compared to conventional 2D *in vitro* cultures, both regarding the arrangement of the cancer cells (e.g. cluster formation *in vivo* and in the 3D model) and their localization (e.g. close proximity to endothelial cells and osteoclasts). Focusing on the design of a bone marrow model, Glaser and co-authors included bone marrow MSCs, osteoblasts, hematopoietic stem/progenitor cells (HSPCs) as well as endothelial cells in a fibrin gel (121). In addition to a different proliferative and invasive behavior of breast cancer cells in their 3D coculture model compared to a fibrin-only control, the authors also observed an altered reaction of the microenvironment to Doxorubicin (i.e. mimicking the neutropenic effect of chemotherapy and increasing neutrophil production, both usually observed *in vivo* upon chemotherapy treatment). This effect was not observed in 2D culture. Unfortunately, the authors did not study the effect of chemotherapy on breast cancer cells in their model. 3D models can also be a great tool to study the molecular pathways leading to drug resistance. For example, understanding how dormancy is induced in breast cancer cells would be of great help to explore therapeutic solutions to avoid breast cancer relapse and late recurrence. With that goal in mind, Pradhan et al. designed a 3D *in vitro* model composed of breast cancer cells, human MSCs and fetal osteoblasts, and demonstrated that cytokines secreted by osteoblasts (e.g. TNFα, MCP1) were able to induce dormancy in the cancer cells (39). This mechanism could be reversed by blocking the receptors of these cytokines with monoclonal antibodies.

Other models have been built to replicate the bone or the bone marrow without a specific focus on breast cancer. For example, Ma et al. created a bone model including endothelial cells, MSCs and osteoblasts embedded in a hydrogel to test the resistance of leukemia cells. The study showed that drug resistance of cancer cells was higher in presence of the bone niche (124). A similar model embedding endothelial cells, MSCs and osteoblasts also focused on leukemia analyzing the differences in drug sensitivity in 2D, 3D static and 3D dynamic conditions (125). The authors demonstrated the protective role of the microenvironment by comparing cancer cell response to a chemotherapeutic agent (i.e. the antimetabolite chemotherapeutic drug Ara-C) and showed increased drug resistance due to the presence of the cells and ECM. The authors hypothesize that this observed chemoresistance can mostly be attributed to CXCL12/CXCR4 signaling, but also to direct cell-cell interactions involving vascular cell adhesion molecule 1/very late antigen-4 (VCAM-1/VLA-4). These two pathways lead to the activation of the prosurvival signaling NF-κB. Finally, several 3D models of bone or bone marrow have been created to test the effect of a surrounding niche on the drug sensitivity of multiple myeloma (126, 127), prostate cancer (128), osteosarcoma (129) and Ewing sarcoma (130, 131). All these models highlighted the requirement of a higher dose of anti-cancer drug to reach the mortality achieved in 2D controls. This effect is mainly due to the microenvironment, either physically preventing the drug to reach cancer cells or by diminishing drug efficacy. These models could be easily adapted to study breast cancer metastases.

Taken together, these examples clearly show that a 2D assay of chemotherapy alone cannot accurately predict the efficacy of the drug in *in vivo* setting, leading to potential drug failure in clinical trial or increased side effects. 3D models with complex microenvironments thus seem to be a better alternative in order to properly replicate the mechanisms involved in cancer proliferation and drug resistance, and would benefit from being developed further. Indeed, multiple cell types such as immune cells, that are now known to have an effect on cancer aggressiveness and drug resistance, have never been tested in a drug screening scenario in a 3D setting. It is likely that introducing them within the biofabricated microenvironment could yield results regarding drug effectiveness better mimicking what is observed in patients.

However, it is important to remember that current *in vitro* 3D models do not have the ability to fully replace rodent models, as their complexity is highly limited by the amount of different cell types able to co-exist in the same matrix, as well as by their simplified architecture that can only partially mimic a real tissue unit. Moreover, off target cytotoxicity cannot be easily tested with 3D models as it would require the development of bodies-on-a-chip [the reader can refer to these reviews for more information about these systems (132, 133)] that are extremely challenging to develop and run. At the same time, even though this and other reviews describe in length the advantages of testing drug compounds in 3D systems, it is worth noting that these models are still thought to be complicated and costly, making them less attractive than 2D monocultures in a high-throughput context (134). However, as technological advances progress and commercially available tests are developed, their cost is expected to become more competitive. Cost reduction and superior performances are expected to outperform conventional 2D screening technologies, especially in cancer research (135, 136).

## 5 Conclusion and perspectives

As highlighted in this review, the microenvironment is a key driver for the establishment of drug resistance in cancer metastases. Unfortunately, the complexity of this microenvironment implies that the combined action of many cell types on drug sensitivity cannot be studied using conventional assays. Advanced *in vitro* 3D models could be a great tool to better understand the mechanisms and pathways involved in the onset of drug resistance and to develop more effective therapeutic options.

Considering the bone microenvironment, multiple cell types influence the behavior of cancer cells when subject to a drug treatment. By protecting cancer cells from apoptosis, guiding them towards dormancy, or even metabolizing the drug themselves, cells from the bone microenvironment put in place a plethora of different mechanisms to help cancer cells to survive chemo-, immune-, radio- or endocrine therapy. Combined with the very high failure rate of anti-cancer drugs during clinical trials, it is becoming clear that the current gold-standards of preclinical testing (i.e. 2D cultures and animal models) are lacking critical information to accurately predict how the drug will behave in the patient. The emergence of more complex 3D models which more accurately mimic cell-ECM and cell-cell interactions thus seems to be a promising alternative (10). However, to this date most bone or bone marrow models are still focusing on the biofabrication of the scaffold and the microenvironment and very few have tried to add cancer cells to their model (137). Noteworthy, only a small fraction of them is focusing on drug resistance. These models have demonstrated that adding a chemotherapy treatment against cancer cells clearly show the establishment of drug resistance induced by the microenvironment (114, 115, 119, 120). This effect is not observed in 2D or monoculture assays.

Regarding breast cancer metastases to bone, it would be extremely helpful to create a comprehensive summary of which cells in the bone microenvironment have an influence on cancer cell proliferation and drug sensitivity. Compiling this summary would require the setup of large parametric studies combining statistical approaches of design of experiment with artificial intelligence-driven data analysis. Having such information would allow to create physiological-like *in vitro* 3D bone models that have the best chance of mimicking accurate cell-cell interactions and identify molecular pathways involved in drug resistance, ultimately helping life-changing treatments to arrive quicker on the market. In particular, these advanced models could help to observe how the bone microenvironment is gradually skewed by cancer cells to shield them from the effect of a given drug.

These models could also be used to test already approved drugs, either for a different bone pathology or as a combination to tackle breast cancer drug resistance. For instance, second line treatments are administered when the first line has failed causing the tumor to start its growth again due to the presence of cells fully resistant to the first treatment (106). With this in mind, administering different chemotherapies as a sequence or a combo before the tumor has time to start growing back could be the future to prevent drug resistance. Studying the onset of unknown drug resistance by testing different treatments in a row could also be a useful technique to find ways to counteract it. Human *in vitro* 3D bone models reproducing the metastatic microenvironment would be an effective tool to perform these studies, potentially employing patient-derived cells in the context of personalized medicine. These models could be easily employed for large-scale drug screenings that cannot be easily performed with *in vivo* models like rodents due to higher costs, raising ethical issues and longer experimental times. Within the drug-discovery pipeline, using 3D *in vitro* bone models as a tool for accurate large-scale drug screening before testing them in animals could help identifying which drugs are expected to yield better results, thus leading to a reduction of animals needed in agreement with the 3Rs principle [replace, reduce, refine (138)]. We thus envision that the integration of the 3D human bone microenvironment with high-throughput drug screening methods would allow for a more efficient pre-clinical testing and improve the success rate of clinical trials.

It is worth mentioning that, while 2D models can only be ameliorated by adding additional cell types to the culture, 3D models present a greater ability to get improved in order to more closely resemble the organ they are mimicking. For example, while cellular complexity seems to be essential to accurately represent mechanisms happening between the cells of interest (e.g. cancer cells) and their microenvironment, there can be many other ways to improve the accuracy of the model. Incorporating a scaffold, inorganic components present in the tissue, flow or even mechanical or electrical simulation will make the model more relevant. This additional level of complexity cannot be included in conventional 2D assays. Regarding the bone, a tissue that is highly vascularized and experiences constant compression forces, introducing flow in a microfluidic device as well as mechanical stimulation would be a great way to better mimic physiological conditions. Indeed, the presence of a flow influences the behavior of bone cells, notably by inducing the release of osteogenic factors which influence the bone remodeling process (i.e. matrix mineralization and collagen deposition, as well as osteoblast proliferation) (139). A similar observation can be made when adding a scaffold whose geometry closely mimics the one found in human tissue units. For instance, the pattern of the scaffold can influence the distribution of shear stress forces as well as bone cell behavior (140). The rest of the microenvironment can also benefit from being cultured in 3D compared to 2D monolayers. For example, bone marrow adipocytes tend to adopt a more *in vivo* like morphology and biochemical behavior in 3D compared to a 2D control. Indeed, cells in a monolayer tend to be forced into a restrictive demeanor with a forced polarity due to the presence of altered focal adhesions. In addition, cells grown on plastic surfaces are subject to an increased stiffness that usually leads to an improper gene expression (141, 142), while cells cultivated on a 3D scaffold show higher survival, differentiation as well as drug sensitivity (141).

Concluding, the field of pre-clinical assays is starting to shift towards more accurate *in vitro* 3D models to predict the efficiency of a drug. Human 3D models of the bone microenvironment have the potential to help understanding in more depth the biological mechanisms underlying drug resistance due to the possibility to selectively introduce specific components (e.g. cells, matrix proteins, growth factors) in the biofabricated model. By balancing complexity and throughput, these systems could be designed to perform large experimental campaigns of drug screening. On a longer time scale, introducing patient-derived cells from biopsies of the metastatic bone tissue would allow to create personalized treatments based on the genetic background of the tumor and on the response of the microenvironment. Finally, designing a tunable microenvironment to fit the specificities of the patient (e.g. introducing pre-existing comorbidities) could further improve the design of patient-specific treatments.

## Author contributions

AL conceptualized and wrote the manuscript. SB and MM reviewed the manuscript. All authors contributed to the article and approved the submitted version.

## References

[1] Martei YM Pace LE Brock JE Shulman LN. Breast cancer in low- and middle-income countries. Clin Lab Med (2018) 38(1):161–73. doi: 10.1016/j.cll.2017.10.013 PMC6277976 29412880

[2] Yedjou CG Sims JN Miele L Noubissi F Lowe L Fonseca DD. Health and racial disparity in breast cancer. Adv Exp Med Biol (2019) 1152:31–49. doi: 10.1007/978-3-030-20301-6_3 31456178 PMC6941147

[3] Yoo GJ Levine EG Pasick R. Breast cancer and coping among women of color: a systematic review of the literature. Support Care Cancer (2014) 22(3):811–24. doi: 10.1007/s00520-013-2057-3 PMC4537653 24389825

[4] Salvador F Llorente A Gomis RR. From latency to overt bone metastasis in breast cancer: potential for treatment and prevention. J Pathol (2019) 249(1):6–18. doi: 10.1002/path.5292 31095738 PMC6771808

[5] Huang JF Shen J Li X Rengan R Silvestris N Wang M. Incidence of patients with bone metastases at diagnosis of solid tumors in adults: a large population-based study. Ann Transl Med (2020) 8(7):482. doi: 10.21037/atm.2020.03.55 32395526 PMC7210217

[6] Zhang W Bado IL Hu J Wan YW Wu L Wang H. The bone microenvironment invigorates metastatic seeds for further dissemination. Cell (2021) 184(9):2471–2486.e20. doi: 10.1016/j.cell.2021.03.011 33878291 PMC8087656

[7] Venetis K Piciotti R Sajjadi E Invernizzi M Morganti S Criscitiello C. Breast cancer with bone metastasis: Molecular insights and clinical management. Cells (2021) 10(6):1377. doi: 10.3390/cells10061377 34199522 PMC8229615

[8] Brook N Brook E Dharmarajan A Dass CR Chan A. Breast cancer bone metastases: pathogenesis and therapeutic targets. Int J Biochem Cell Biol (2018) 96:63–78. doi: 10.1016/j.biocel.2018.01.003 29309917

[9] Massagué J Obenauf AC. Metastatic colonization. Nature (2016) 529(7586):298–306. doi: 10.1038/nature17038 26791720 PMC5029466

[10] Moffat JG Vincent F Lee JA Eder J Prunotto M. Opportunities and challenges in phenotypic drug discovery: an industry perspective. Nat Rev Drug Discovery (2017) 16(8):531–43. doi: 10.1038/nrd.2017.111 28685762

[11] Mestas J Hughes CCW. Of mice and not men: Differences between mouse and human immunology. J Immunol (2004) 172(5):2731–8. doi: 10.4049/jimmunol.172.5.2731 14978070

[12] Rosol TJ Tannehill-Gregg SH LeRoy BE Mandl S Contag CH. Animal models of bone metastasis. Cancer Treat Res (2004) 118:47–81. doi: 10.1007/978-1-4419-9129-4_3 15043188 PMC3057671

[13] Mittal R Woo FW Castro CS Cohen MA Karanxha J Mittal J. Organ-on-chip models: Implications in drug discovery and clinical applications. J Cell Physiol (2019) 234(6):8352–80. doi: 10.1002/jcp.27729 30443904

[14] Jubelin C Muñoz-Garcia J Griscom L Cochonneau D Ollivier E Heymann MF. Three-dimensional *in vitro* culture models in oncology research. Cell Biosci (2022) 12(1):155. doi: 10.1186/s13578-022-00887-3 36089610 PMC9465969

[15] Hoarau-Véchot J Rafii A Touboul C Pasquier J. Halfway between 2D and animal models: Are 3D cultures the ideal tool to study cancer-microenvironment interactions? Int J Mol Sci (2018) 19(1):181. doi: 10.3390/ijms19010181 29346265 PMC5796130

[16] Langhans SA. Three-dimensional in vitro cell culture models in drug discovery and drug repositioning. Front Pharmacol (2018) 9:6. doi: 10.3389/fphar.2018.00006 29410625 PMC5787088

[17] Ewart L Apostolou A Briggs SA Carman CV Chaff JT Heng AR. Performance assessment and economic analysis of a human liver-chip for predictive toxicology. Commun Med (2022) 2(1):1–16. doi: 10.1038/s43856-022-00209-1 36473994 PMC9727064

[18] Akhtari M Mansuri J Newman KA Guise TM Seth P. Biology of breast cancer bone metastasis. Cancer Biol Ther (2008) 7(1):3–9. doi: 10.4161/cbt.7.1.5163 18059174

[19] Liu Y Cao X. Characteristics and significance of the pre-metastatic niche. Cancer Cell (2016) 30(5):668–81. doi: 10.1016/j.ccell.2016.09.011 27846389

[20] Sanmartin MC Borzone FR Giorello MB Pacienza N Yannarelli G Chasseing NA. Bone marrow/bone pre-metastatic niche for breast cancer cells colonization: The role of mesenchymal stromal cells. Crit Rev Oncol Hematol (2021) 164:103416. doi: 10.1016/j.critrevonc.2021.103416 34237436

[21] Zhang Y Ma B Fan Q. Mechanisms of breast cancer bone metastasis. Cancer Lett (2010) 292(1):1–7. doi: 10.1016/j.canlet.2009.11.003 20006425

[22] Mukaida N Zhang D Sasaki SI. Emergence of cancer-associated fibroblasts as an indispensable cellular player in bone metastasis process. Cancers (Basel) (2020) 12(10):E2896. doi: 10.3390/cancers12102896 PMC7600711 33050237

[23] Le Pape F Vargas G Clézardin P. The role of osteoclasts in breast cancer bone metastasis. J Bone Oncol (2016) 5(3):93–5. doi: 10.1016/j.jbo.2016.02.008 PMC5063222 27761364

[24] Treatment options. Available at: https://www.breastcancer.org/treatment.

[25] Chemotherapy for breast cancer | breast cancer treatment. Available at: https://www.cancer.org/cancer/breast-cancer/treatment/chemotherapy-for-breast-cancer.html.

[26] Goswami S Chen Y Anandhan S Szabo PM Basu S Blando JM. ARID1A mutation plus CXCL13 expression act as combinatorial biomarkers to predict responses to immune checkpoint therapy in mUCC. Sci Trans Med (2020) 12(548):eabc4220. doi: 10.1126/scitranslmed.abc4220 32554706

[27] Twomey JD Zhang B. Cancer immunotherapy update: FDA-approved checkpoint inhibitors and companion diagnostics. AAPS J (2021) 23(2):39. doi: 10.1208/s12248-021-00574-0 33677681 PMC7937597

[28] Darvin P Toor SM Sasidharan Nair V Elkord E. Immune checkpoint inhibitors: recent progress and potential biomarkers. Exp Mol Med (2018) 50(12):1–11. doi: 10.1038/s12276-018-0191-1 PMC6292890 30546008

[29] Mellman I Coukos G Dranoff G. Cancer immunotherapy comes of age. Nature (2011) 480(7378):480–9. doi: 10.1038/nature10673 PMC3967235 22193102

[30] Liu C Wang M Xu C Li B Chen J Chen J. Immune checkpoint inhibitor therapy for bone metastases: Specific microenvironment and current situation. J Immunol Res (2021) 2021:e8970173. doi: 10.1155/2021/8970173 PMC8645368 34877360

[31] Chemotherapy’s effects on organ and body systems - health encyclopedia - university of Rochester medical center. Available at: https://www.urmc.rochester.edu/encyclopedia/content.aspx?ContentID=P07155&ContentTypeID=85.

[32] Side effects of immunotherapy - NCI. Available at: https://www.cancer.gov/about-cancer/treatment/types/immunotherapy/side-effects.

[33] Martins F Sofiya L Sykiotis GP Lamine F Maillard M Fraga M. Adverse effects of immune-checkpoint inhibitors: epidemiology, management and surveillance. Nat Rev Clin Oncol (2019) 16(9):563–80. doi: 10.1038/s41571-019-0218-0 31092901

[34] Rizzoli R Ferrari S. Bisphosphonates and combined treatments osteoporosis. Rev Med Suisse (2005) 1(35):2269–71. 16268449

[35] Chen J Zhou L Liu X Wen X Li H Li W. Meta-analysis of clinical trials to assess denosumab over zoledronic acid in bone metastasis. Int J Clin Pharm (2021) 43(1):2–10. doi: 10.1007/s11096-020-01105-1 32964403

[36] Tsoi H Man EPS Chau KM Khoo US. Targeting the IL-6/STAT3 signalling cascade to reverse tamoxifen resistance in estrogen receptor positive breast cancer. Cancers (Basel) (2021) 13(7):1511. doi: 10.3390/cancers13071511 33806019 PMC8036560

[37] Bado IL Zhang W Hu J Xu Z Wang H Sarkar P. The bone microenvironment increases phenotypic plasticity of ER+ breast cancer cells. Dev Cell (2021) 56(8):1100–1117.e9. doi: 10.1016/j.devcel.2021.03.008 33878299 PMC8062036

[38] Zheng H Bae Y Kasimir-Bauer S Tang R Chen J Ren G. Therapeutic antibody targeting tumor- and osteoblastic niche-derived Jagged1 sensitizes bone metastasis to chemotherapy. Cancer Cell (2017) 32(6):731–747.e6. doi: 10.1016/j.ccell.2017.11.002 29232552 PMC5729937

[39] Pradhan L Moore D Ovadia EM Swedzinski SL Cossette T Sikes RA. Dynamic bioinspired coculture model for probing ER+ breast cancer dormancy in the bone marrow niche. Sci Adv (2023) 9(10):eade3186. doi: 10.1126/sciadv.ade3186 36888709 PMC9995072

[40] Portillo-Lara R Annabi N. Microengineered cancer-on-a-chip platforms to study the metastatic microenvironment. Lab Chip (2016) 16(21):4063–81. doi: 10.1039/C6LC00718J 27605305

[41] Chauhan VP Martin JD Liu H Lacorre DA Jain SR Kozin SV. Angiotensin inhibition enhances drug delivery and potentiates chemotherapy by decompressing tumour blood vessels. Nat Commun (2013) 4:2516. doi: 10.1038/ncomms3516 24084631 PMC3806395

[42] Shintani Y Fujiwara A Kimura T Kawamura T Funaki S Minami M. IL-6 secreted from cancer-associated fibroblasts mediates chemoresistance in NSCLC by increasing epithelial-mesenchymal transition signaling. J Thorac Oncol (2016) 11(9):1482–92. doi: 10.1016/j.jtho.2016.05.025 27287412

[43] Bussard KM Mutkus L Stumpf K Gomez-Manzano C Marini FC. Tumor-associated stromal cells as key contributors to the tumor microenvironment. Breast Cancer Res (2016) 18(1):84. doi: 10.1186/s13058-016-0740-2 27515302 PMC4982339

[44] Xiao M He J Yin L Chen X Zu X Shen Y. Tumor-associated macrophages: Critical players in drug resistance of breast cancer. Front Immunol (2021) 12:799428. doi: 10.3389/fimmu.2021.799428 34992609 PMC8724912

[45] Walker ND Elias M Guiro K Bhatia R Greco SJ Bryan M. Exosomes from differentially activated macrophages influence dormancy or resurgence of breast cancer cells within bone marrow stroma. Cell Death Dis (2019) 10(2):1–16. doi: 10.1038/s41419-019-1304-z PMC6347644 30683851

[46] Chesney JA Mitchell RA Yaddanapudi K. Myeloid-derived suppressor cells-a new therapeutic target to overcome resistance to cancer immunotherapy. J Leukoc Biol (2017) 102(3):727–40. doi: 10.1189/jlb.5VMR1116-458RRR PMC6608049 28546500

[47] Acharyya S Oskarsson T Vanharanta S Malladi S Kim J Morris PG. A CXCL1 paracrine network links cancer chemoresistance and metastasis. Cell (2012) 150(1):165–78. doi: 10.1016/j.cell.2012.04.042 PMC3528019 22770218

[48] Sato K Ozaki K Oh I Meguro A Hatanaka K Nagai T. Nitric oxide plays a critical role in suppression of T-cell proliferation by mesenchymal stem cells. Blood (2006) 109(1):228–34. doi: 10.1182/blood-2006-02-002246 16985180

[49] Lim PK Bliss SA Patel SA Taborga M Dave MA Gregory LA. Gap junction–mediated import of MicroRNA from bone marrow stromal cells can elicit cell cycle quiescence in breast cancer cells. Cancer Res (2011) 71(5):1550–60. doi: 10.1158/0008-5472.CAN-10-2372 21343399

[50] Bodenstine TM Vaidya KS Ismail A Beck BH Cook LM Diers AR. Homotypic gap junctional communication associated with metastasis suppression increases with PKA activity and is unaffected by PI3K inhibition. Cancer Res (2010) 70(23):10002–11. doi: 10.1158/0008-5472.CAN-10-2606 PMC3003438 21098703

[51] Roodhart JML Daenen LGM Stigter ECA Prins HJ Gerrits J Houthuijzen JM. Mesenchymal stem cells induce resistance to chemotherapy through the release of platinum-induced fatty acids. Cancer Cell (2011) 20(3):370–83. doi: 10.1016/j.ccr.2011.08.010 21907927

[52] Zheng H Li W Kang Y. Tumor-stroma interactions in bone metastasis: Molecular mechanisms and therapeutic implications. Cold Spring Harb Symp Quant Biol (2016) 81:151–61. doi: 10.1101/sqb.2016.81.030775 28381439

[53] Samimi A Ghanavat M Shahrabi S Azizidoost S Saki N. Role of bone marrow adipocytes in leukemia and chemotherapy challenges. Cell Mol Life Sci (2019) 76(13):2489–97. doi: 10.1007/s00018-019-03031-6 PMC11105633 30715556

[54] Lehuédé C Li X Dauvillier S Vaysse C Franchet C Clement E. Adipocytes promote breast cancer resistance to chemotherapy, a process amplified by obesity: role of the major vault protein (MVP). Breast Cancer Res (2019) 21(1):7. doi: 10.1186/s13058-018-1088-6 30654824 PMC6337862

[55] Carlson P Dasgupta A Grzelak CA Kim J Barrett A Coleman IM. Targeting the perivascular niche sensitizes disseminated tumour cells to chemotherapy. Nat Cell Biol (2019) 21(2):238–50. doi: 10.1038/s41556-018-0267-0 PMC6948102 30664790

[56] Hoyt DG Rusnak JM Mannix RJ Modzelewski RA Johnson CS Lazo JS. Integrin activation suppresses etoposide-induced DNA strand breakage in cultured murine tumor-derived endothelial cells. Cancer Res (1996) 56(18):4146–9. 8797583

[57] Aoudjit F Vuori K. Integrin signaling inhibits paclitaxel-induced apoptosis in breast cancer cells. Oncogene (2001) 20(36):4995–5004. doi: 10.1038/sj.onc.1204554 11526484

[58] Yin JJ Pollock CB Kelly K. Mechanisms of cancer metastasis to the bone. Cell Res (2005) 15(1):57–62. doi: 10.1038/sj.cr.7290266 15686629

[59] Wang H Tian L Liu J Goldstein A Bado I Zhang W. The osteogenic niche is a calcium reservoir of bone micrometastases and confers unexpected therapeutic vulnerability. Cancer Cell (2018) 34(5):823–839.e7. doi: 10.1016/j.ccell.2018.10.002 30423299 PMC6239211

[60] Lu Y Zhang J Dai J Dehne LA Mizokami A Yao Z. Osteoblasts induce prostate cancer proliferation and PSA expression through interleukin-6-mediated activation of the androgen receptor. Clin Exp Metastasis (2004) 21(5):399–408. doi: 10.1007/s10585-005-0056-6 15672864

[61] Ping Q Yan R Cheng X Wang W Zhong Y Hou Z. Cancer-associated fibroblasts: overview, progress, challenges, and directions. Cancer Gene Ther (2021) 28(9):984–99. doi: 10.1038/s41417-021-00318-4 33712707

[62] Sahai E Astsaturov I Cukierman E DeNardo DG Egeblad M Evans RM. A framework for advancing our understanding of cancer-associated fibroblasts. Nat Rev Cancer (2020) 20(3):174–86. doi: 10.1038/s41568-019-0238-1 PMC7046529 31980749

[63] Mao X Xu J Wang W Liang C Hua J Liu J. Crosstalk between cancer-associated fibroblasts and immune cells in the tumor microenvironment: new findings and future perspectives. Mol Cancer (2021) 20:131. doi: 10.1186/s12943-021-01428-1 34635121 PMC8504100

[64] Chan JSK Sng MK Teo ZQ Chong HC Twang JS Tan NS. Targeting nuclear receptors in cancer-associated fibroblasts as concurrent therapy to inhibit development of chemoresistant tumors. Oncogene (2018) 37(2):160–73. doi: 10.1038/onc.2017.319 PMC5770601 28892046

[65] Haider MT Smit DJ Taipaleenmäki H. The endosteal niche in breast cancer bone metastasis. Front Oncol (2020) 10:335. doi: 10.3389/fonc.2020.00335 32232008 PMC7082928

[66] Jain RK Martin JD Stylianopoulos T. The role of mechanical forces in tumor growth and therapy. Annu Rev BioMed Eng (2014) 16:321–46. doi: 10.1146/annurev-bioeng-071813-105259 PMC4109025 25014786

[67] Peiris-Pagès M Sotgia F Lisanti MP. Chemotherapy induces the cancer-associated fibroblast phenotype, activating paracrine hedgehog-GLI signalling in breast cancer cells. Oncotarget (2015) 6(13):10728–45. doi: 10.18632/oncotarget.3828 PMC4484415 25915429

[68] Belli C Trapani D Viale G D’Amico P Duso BA Della Vigna P. Targeting the microenvironment in solid tumors. Cancer Treat Rev (2018) 65:22–32. doi: 10.1016/j.ctrv.2018.02.004 29502037

[69] Mendoza-Reinoso V McCauley LK Fournier PGJ. Contribution of macrophages and T cells in skeletal metastasis. Cancers (Basel) (2020) 12(4):E1014. doi: 10.3390/cancers12041014 PMC7226332 32326073

[70] Strachan DC Ruffell B Oei Y Bissell MJ Coussens LM Pryer N. CSF1R inhibition delays cervical and mammary tumor growth in murine models by attenuating the turnover of tumor-associated macrophages and enhancing infiltration by CD8+ T cells. Oncoimmunology (2013) 2(12):e26968. doi: 10.4161/onci.26968 24498562 PMC3902121

[71] Ruffell B Chang-Strachan D Chan V Rosenbusch A Ho CMT Pryer N. Macrophage IL-10 blocks CD8+ T cell-dependent responses to chemotherapy by suppressing IL-12 expression in intratumoral dendritic cells. Cancer Cell (2014) 26(5):623–37. doi: 10.1016/j.ccell.2014.09.006 PMC4254570 25446896

[72] Xu M Zhao Z Song J Lan X Lu S Chen M. Interactions between interleukin-6 and myeloid-derived suppressor cells drive the chemoresistant phenotype of hepatocellular cancer. Exp Cell Res (2017) 351(2):142–9. doi: 10.1016/j.yexcr.2017.01.008 28109867

[73] Felcher CM Bogni ES Kordon EC. IL-6 cytokine family: A putative target for breast cancer prevention and treatment. Int J Mol Sci (2022) 23(3):1809. doi: 10.3390/ijms23031809 35163731 PMC8836921

[74] Ramachandran IR Condamine T Lin C Herlihy SE Garfall A Vogl DT. Bone marrow PMN-MDSCs and neutrophils are functionally similar in protection of multiple myeloma from chemotherapy. Cancer Lett (2016) 371(1):117–24. doi: 10.1016/j.canlet.2015.10.040 PMC4919899 26639197

[75] Patel S Fu S Mastio J Dominguez GA Purohit A Kossenkov A. Unique pattern of neutrophil migration and function during tumor progression. Nat Immunol (2018) 19(11):1236–47. doi: 10.1038/s41590-018-0229-5 PMC6195445 30323345

[76] Saini M Szczerba BM Aceto N. Circulating tumor cell-neutrophil tango along the metastatic process. Cancer Res (2019) 79(24):6067–73. doi: 10.1158/0008-5472.CAN-19-1972 31527091

[77] Wik JA Skålhegg BS. T Cell metabolism in infection. Front Immunol (2022) 13:840610. doi: 10.3389/fimmu.2022.840610 35359994 PMC8964062

[78] Thommen DS Schumacher TN. T Cell dysfunction in cancer. Cancer Cell (2018) 33(4):547–62. doi: 10.1016/j.ccell.2018.03.012 PMC7116508 29634943

[79] Farhood B Najafi M Mortezaee K. CD8+ cytotoxic T lymphocytes in cancer immunotherapy: A review. J Cell Physiol (2019) 234(6):8509–21. doi: 10.1002/jcp.27782 30520029

[80] Kond˘lková K Vokurková D Krejsek J Borská L Fiala Z Ctirad A. Regulatory T cells (TREG) and their roles in immune system with respect to immunopathological disorders. Acta Med (Hradec Kralove) (2010) 53(2):73–7. doi: 10.14712/18059694.2016.63 20672742

[81] Whiteside TL. The tumor microenvironment and its role in promoting tumor growth. Oncogene (2008) 27(45):5904–12. doi: 10.1038/onc.2008.271 PMC3689267 18836471

[82] Xiang P Jin S Yang Y Sheng J He Q Song Y. Infiltrating CD4+ T cells attenuate chemotherapy sensitivity in prostate cancer *via* CCL5 signaling. Prostate (2019) 79(9):1018–31. doi: 10.1002/pros.23810 PMC6594129 31018021

[83] Manore SG Doheny DL Wong GL Lo HW. IL-6/JAK/STAT3 signaling in breast cancer metastasis: Biology and treatment. Front Oncol (2022) 12:866014. doi: 10.3389/fonc.2022.866014 35371975 PMC8964978

[84] Zhao P Xiao L Peng J Qian YQ Huang CC. Exosomes derived from bone marrow mesenchymal stem cells improve osteoporosis through promoting osteoblast proliferation *via* MAPK pathway. Eur Rev Med Pharmacol Sci (2018) 22(12):3962–70. doi: 10.26355/eurrev_201806_15280 29949171

[85] Liu S Liu F Zhou Y Jin B Sun Q Guo S. Immunosuppressive property of MSCs mediated by cell surface receptors. Front Immunol (2020) 11:1076. doi: 10.3389/fimmu.2020.01076 32849489 PMC7399134

[86] Pacini S Petrini I. Are MSCs angiogenic cells? new insights on human nestin-positive bone marrow-derived multipotent cells. Front Cell Dev Biol (2014) 2:20. doi: 10.3389/fcell.2014.00020 25364727 PMC4207020

[87] Ahn SY. The role of MSCs in the tumor microenvironment and tumor progression. Anticancer Res (2020) 40(6):3039–47. doi: 10.21873/anticanres.14284 32487597

[88] Patel SA Ramkissoon SH Bryan M Pliner LF Dontu G Patel PS. Delineation of breast cancer cell hierarchy identifies the subset responsible for dormancy. Sci Rep (2012) 2:906. doi: 10.1038/srep00906 23205268 PMC3510468

[89] Pinho S Frenette PS. Haematopoietic stem cell activity and interactions with the niche. Nat Rev Mol Cell Biol (2019) 20(5):303–20. doi: 10.1038/s41580-019-0103-9 PMC6483843 30745579

[90] Sugiyama T Kohara H Noda M Nagasawa T. Maintenance of the hematopoietic stem cell pool by CXCL12-CXCR4 chemokine signaling in bone marrow stromal cell niches. Immunity (2006) 25(6):977–88. doi: 10.1016/j.immuni.2006.10.016 17174120

[91] Dubrovska A Hartung A Bouchez LC Walker JR Reddy VA Cho CY. CXCR4 activation maintains a stem cell population in tamoxifen-resistant breast cancer cells through AhR signalling. Br J Cancer (2012) 107(1):43–52. doi: 10.1038/bjc.2012.105 22644306 PMC3389396

[92] Mukherjee D Zhao J. The role of chemokine receptor CXCR4 in breast cancer metastasis. Am J Cancer Res (2013) 3(1):46–57. 23359227 PMC3555200

[93] Nobutani K Shimono Y Mizutani K Ueda Y Suzuki T Kitayama M. Downregulation of CXCR4 in metastasized breast cancer cells and implication in their dormancy. PloS One (2015) 10(6):e0130032. doi: 10.1371/journal.pone.0130032 26083776 PMC4470829

[94] Shiozawa Y Pienta KJ Taichman RS. Hematopoietic stem cell niche is a potential therapeutic target for bone metastatic tumors. Clin Cancer Res (2011) 17(17):5553–8. doi: 10.1158/1078-0432.CCR-10-2505 PMC3593121 21676926

[95] Capulli M Hristova D Valbret Z Carys K Arjan R Maurizi A. Notch2 pathway mediates breast cancer cellular dormancy and mobilisation in bone and contributes to haematopoietic stem cell mimicry. Br J Cancer (2019) 121(2):157–71. doi: 10.1038/s41416-019-0501-y PMC6738045 31239543

[96] Liu M Mo QG Wei CY Qin QH Huang Z He J. Platinum-based chemotherapy in triple-negative breast cancer: A meta-analysis. Oncol Lett (2013) 5(3):983–91. doi: 10.3892/ol.2012.1093 PMC3576281 23426861

[97] Aaron N Costa S Rosen CJ Qiang L. The implications of bone marrow adipose tissue on inflammaging. Front Endocrinol (2022) 13:853765. doi: 10.3389/fendo.2022.853765 PMC8962663 35360075

[98] Shin E Koo JS. The role of adipokines and bone marrow adipocytes in breast cancer bone metastasis. Int J Mol Sci (2020) 21(14):4967. doi: 10.3390/ijms21144967 32674405 PMC7404398

[99] The effect of marrow secretome and culture environment on the rate of metastatic breast cancer cell migration in two and three dimensions - PubMed. Available at: https://pubmed.ncbi.nlm.nih.gov/33689396/. 10.1091/mbc.E19-12-0682 PMC8101488 33689396

[100] Zarrer J Haider MT Smit DJ Taipaleenmäki H. Pathological crosstalk between metastatic breast cancer cells and the bone microenvironment. Biomolecules (2020) 10(2):E337. doi: 10.3390/biom10020337 PMC7072692 32092997

[101] Reagan MR Fairfield H Rosen CJ. Bone marrow adipocytes: A link between obesity and bone cancer. Cancers (Basel) (2021) 13(3):364. doi: 10.3390/cancers13030364 33498240 PMC7863952

[102] Hanahan D Coussens LM. Accessories to the crime: functions of cells recruited to the tumor microenvironment. Cancer Cell (2012) 21(3):309–22. doi: 10.1016/j.ccr.2012.02.022 22439926

[103] Raymaekers K Stegen S van Gastel N Carmeliet G. The vasculature: a vessel for bone metastasis. Bonekey Rep (2015) 4:742. doi: 10.1038/bonekey.2015.111 27217954 PMC4859769

[104] Cao Z Ding BS Guo P Lee SB Butler JM Casey SC. Angiocrine factors deployed by tumor vascular niche induce b cell lymphoma invasiveness and chemoresistance. Cancer Cell (2014) 25(3):350–65. doi: 10.1016/j.ccr.2014.02.005 PMC4017921 24651014

[105] Zhang Y Xie ZY Guo XT Xiao XH Xiong LX. Notch and breast cancer metastasis: Current knowledge, new sights and targeted therapy. Oncol Lett (2019) 18(3):2743. doi: 10.3892/ol.2019.10653 31452752 PMC6704289

[106] Gatenby RA Brown JS. Integrating evolutionary dynamics into cancer therapy. Nat Rev Clin Oncol (2020) 17(11):675–86. doi: 10.1038/s41571-020-0411-1 32699310

[107] Karagiannis GS Condeelis JS Oktay MH. Chemotherapy-induced metastasis: mechanisms and translational opportunities. Clin Exp Metastasis (2018) 35(4):269–84. doi: 10.1007/s10585-017-9870-x PMC6035114 29307118

[108] Subia B Dahiya UR Mishra S Ayache J Casquillas GV Caballero D. Breast tumor-on-chip models: From disease modeling to personalized drug screening. J Control Release (2021) 331:103–20. doi: 10.1016/j.jconrel.2020.12.057 PMC8172385 33417986

[109] Gonçalves IM Carvalho V Rodrigues RO Pinho D Teixeira SFCF Moita A. Organ-on-a-Chip platforms for drug screening and delivery in tumor cells: A systematic review. Cancers (Basel) (2022) 14(4):935. doi: 10.3390/cancers14040935 35205683 PMC8870045

[110] Sun W Luo Z Lee J Kim HJ Lee K Tebon P. Organ-on-a-Chip for cancer and immune organs modeling. Adv Healthc Mater (2019) 8(4):e1801363. doi: 10.1002/adhm.201801363 30605261 PMC6424124

[111] Bersini S Arrigoni C Lopa S Bongio M Martin I Moretti M. Engineered miniaturized models of musculoskeletal diseases. Drug Discovery Today (2016) 21(9):1429–36. doi: 10.1016/j.drudis.2016.04.015 27132520

[112] Kim C Bang JH Kim YE Lee SH Kang JY. On-chip anticancer drug test of regular tumor spheroids formed in microwells by a distributive microchannel network. Lab Chip (2012) 12(20):4135–42. doi: 10.1039/c2lc40570a 22864534

[113] Brancato V Gioiella F Imparato G Guarnieri D Urciuolo F Netti PA. 3D breast cancer microtissue reveals the role of tumor microenvironment on the transport and efficacy of free-doxorubicin in vitro. Acta Biomater (2018) 75:200–12. doi: 10.1016/j.actbio.2018.05.055 29864516

[114] Zhu W Holmes B Glazer RI Zhang LG. 3D printed nanocomposite matrix for the study of breast cancer bone metastasis. Nanomed: Nanotechnol Biol Med (2016) 12(1):69–79. doi: 10.1016/j.nano.2015.09.010 26472048

[115] Kar S Katti DR Katti KS. Bone interface modulates drug resistance in breast cancer bone metastasis. Colloids Surfaces B: Biointerfaces (2020) 195:111224. doi: 10.1016/j.colsurfb.2020.111224 32634713

[116] Sharifi S Barar J Hejazi MS Samadi N. Roles of the bcl-2/Bax ratio, caspase-8 and 9 in resistance of breast cancer cells to paclitaxel. Asian Pac J Cancer Prev (2014) 15(20):8617–22. doi: 10.7314/APJCP.2014.15.20.8617 25374178

[117] Litviakov NV Cherdyntseva NV Tsyganov MM Denisov EV Garbukov EY Merzliakova MK. Changing the expression vector of multidrug resistance genes is related to neoadjuvant chemotherapy response. Cancer Chemother Pharmacol (2013) 71(1):153–63. doi: 10.1007/s00280-012-1992-x 23053273

[118] Li YJ Lei YH Yao N Wang CR Hu N Ye WC. Autophagy and multidrug resistance in cancer. Chin J Cancer (2017) 36(1):52. doi: 10.1186/s40880-017-0219-2 28646911 PMC5482965

[119] Langer EM Allen-Petersen BL King SM Kendsersky ND Turnidge MA Kuziel GM. Modeling tumor phenotypes in vitro with three-dimensional bioprinting. Cell Rep (2019) 26(3):608–623.e6. doi: 10.1016/j.celrep.2018.12.090 30650355 PMC6366459

[120] Marturano-Kruik A Nava MM Yeager K Chramiec A Hao L Robinson S. Human bone perivascular niche-on-a-chip for studying metastatic colonization. Proc Natl Acad Sci (2018) 115(6):1256–61. doi: 10.1073/pnas.1714282115 PMC5819403 29363599

[121] Glaser DE Curtis MB Sariano PA Rollins ZA Shergill BS Anand A. Organ-on-a-chip model of vascularized human bone marrow niches. Biomaterials (2022) 280:121245. doi: 10.1016/j.biomaterials.2021.121245 34810038 PMC10658812

[122] Crippa M Talò G Lamouline A Bolis S Arrigoni C Bersini S. A microfluidic model of human vascularized breast cancer metastasis to bone for the study of neutrophil-cancer cell interactions. Mater Today Bio (2022) 17:100460. doi: 10.1016/j.mtbio.2022.100460 PMC9583110 36278146

[123] Colombo MV Bersini S Arrigoni C Gilardi M Sansoni V Ragni E. Engineering the early bone metastatic niche through human vascularized immuno bone minitissues. Biofabrication (2021) 13(3):035036. doi: 10.1088/1758-5090/abefea 33735854

[124] Ma C Witkowski MT Harris J Dolgalev I Sreeram S Qian W. Leukemia-on-a-chip: Dissecting the chemoresistance mechanisms in b cell acute lymphoblastic leukemia bone marrow niche. Sci Adv (2020) 6(44):eaba5536. doi: 10.1126/sciadv.aba5536 33127669 PMC7608809

[125] Bruce A Evans R Mezan R Shi L Moses BS Martin KH. Three-dimensional microfluidic tri-culture model of the bone marrow microenvironment for study of acute lymphoblastic leukemia. PloS One (2015) 10(10):e0140506. doi: 10.1371/journal.pone.0140506 26488876 PMC4619215

[126] Khin ZP Ribeiro MLC Jacobson T Hazlehurst L Perez L Baz R. A preclinical assay for chemosensitivity in multiple myeloma. Cancer Res (2014) 74(1):56–67. doi: 10.1158/0008-5472.CAN-13-2397 24310398 PMC3915502

[127] Pak C Callander NS Young EWK Titz B Kim K Saha S. MicroC3: an ex vivo microfluidic cis-coculture assay to test chemosensitivity and resistance of patient multiple myeloma cells. Integr Biol (Camb) (2015) 7(6):643–54. doi: 10.1039/C5IB00071H PMC4476551 25998180

[128] Fitzgerald KA Guo J Tierney EG Curtin CM Malhotra M Darcy R. The use of collagen-based scaffolds to simulate prostate cancer bone metastases with potential for evaluating delivery of nanoparticulate gene therapeutics. Biomaterials (2015) 66:53–66. doi: 10.1016/j.biomaterials.2015.07.019 26196533

[129] Rimann M Laternser S Gvozdenovic A Muff R Fuchs B Kelm JM. An *in vitro* osteosarcoma 3D microtissue model for drug development. J Biotechnol (2014) 189:129–35. doi: 10.1016/j.jbiotec.2014.09.005 25234575

[130] Fong ELS Lamhamedi-Cherradi SE Burdett E Ramamoorthy V Lazar AJ Kasper FK. Modeling Ewing sarcoma tumors *in vitro* with 3D scaffolds. Proc Natl Acad Sci U S A (2013) 110(16):6500–5. doi: 10.1073/pnas.1221403110 PMC3631678 23576741

[131] Santoro M Menegaz BA Lamhamedi-Cherradi SE Molina ER Wu D Priebe W. Modeling stroma-induced drug resistance in a tissue-engineered tumor model of Ewing sarcoma. Tissue Eng Part A (2017) 23(1–2):80–9. doi: 10.1089/ten.tea.2016.0369 PMC5240012 27923328

[132] Picollet-D’hahan N Zuchowska A Lemeunier I Le Gac S. Multiorgan-on-a-Chip: A systemic approach to model and decipher inter-organ communication. Trends Biotechnol (2021) 39(8):788–810. doi: 10.1016/j.tibtech.2020.11.014 33541718

[133] Sung JH Wang YI Sriram NN Jackson M Long C Hickman JJ. Recent advances in body-on-a-chip systems. Anal Chem (2019) 91(1):330–51. doi: 10.1021/acs.analchem.8b05293 PMC6687466 30472828

[134] 2D vs 3D - creative biolabs. Available at: https://www.creative-biolabs.com/adc/2d-vs-3d.htm.

[135] Jensen C Teng Y. Is it time to start transitioning from 2D to 3D cell culture? Front Mol Biosci (2020) 7:33. doi: 10.3389/fmolb.2020.00033 32211418 PMC7067892

[136] Fontoura JC Viezzer C dos Santos FG Ligabue RA Weinlich R Puga RD. Comparison of 2D and 3D cell culture models for cell growth, gene expression and drug resistance. Mater Sci Engineering: C (2020) 107:110264. doi: 10.1016/j.msec.2019.110264 31761183

[137] Fischetti T Di Pompo G Baldini N Avnet S Graziani G. 3D printing and bioprinting to model bone cancer: The role of materials and nanoscale cues in directing cell behavior. Cancers (Basel) (2021) 13(16):4065. doi: 10.3390/cancers13164065 34439218 PMC8391202

[138] Hampshire VA Gilbert SH. Refinement, reduction, and replacement (3R) strategies in preclinical testing of medical devices. Toxicol Pathol (2019) 47(3):329–38. doi: 10.1177/0192623318797289 30270765

[139] Wittkowske C Reilly GC Lacroix D Perrault CM. *In vitro* bone cell models: Impact of fluid shear stress on bone formation. Front Bioengineering Biotechnol (2016) 4:87. doi: 10.3389/fbioe.2016.00087 PMC5108781 27896266

[140] Mainardi VL Arrigoni C Bianchi E Talò G Delcogliano M Candrian C. Improving cell seeding efficiency through modification of fiber geometry in 3D printed scaffolds. Biofabrication (2021) 13(3):035025. doi: 10.1088/1758-5090/abe5b4 33578401

[141] Lewis KT MacDougald OA. Bone: Bone marrow adipocytes in 3D. Nat Rev Endocrinol (2018) 14(5):254–5. doi: 10.1038/nrendo.2018.31 PMC6986365 29546873

[142] Yi B Xu Q Liu W. An overview of substrate stiffness guided cellular response and its applications in tissue regeneration. Bioactive Mater (2022), 15: 82–102. doi: 10.1016/j.bioactmat.2021.12.005 PMC8940767 35386347

